# Model-Based Investigation of the Relationship between Regulation Level and Pulse Property of I1-FFL Gene Circuits

**DOI:** 10.1021/acssynbio.2c00109

**Published:** 2022-06-22

**Authors:** Jordan Ryan, Seongho Hong, Mathias Foo, Jongmin Kim, Xun Tang

**Affiliations:** † Cain Department of Chemical Engineering, Louisiana State University, Baton Rouge, Louisiana 70803, United States; ‡ School of Engineering, University of Warwick, Coventry CV4 7AL, United Kingdom; ¶ Department of Life Sciences, Pohang University of Science and Technology (POSTECH), Pohang, Gyeongbuk 37673, South Korea

**Keywords:** incoherent feed-forward loop, mathematical modeling, RNA technology

## Abstract

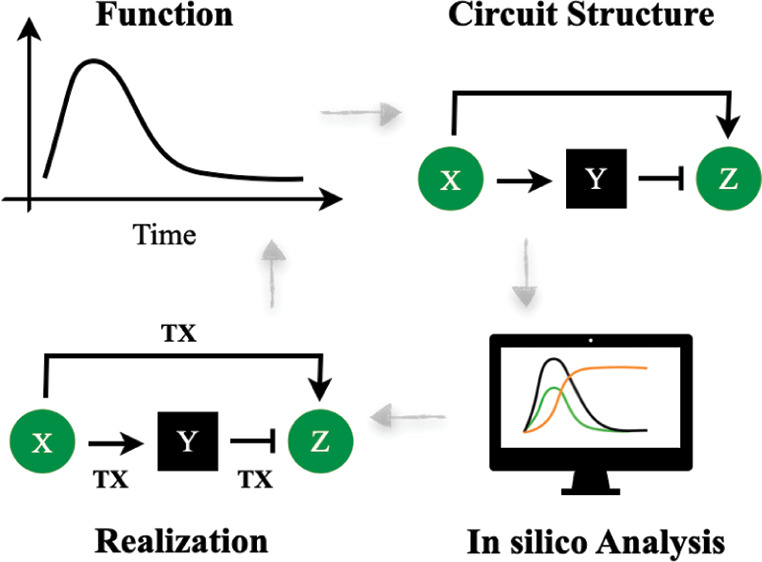

Mathematical models are powerful tools in guiding the construction of synthetic biological circuits, given their capability of accurately capturing and predicting circuit dynamics. Recent innovations in RNA technology have enabled the development of a variety of new tools for regulating gene expression at both the transcription and translation levels. However, the effects of different regulation levels on the circuit dynamics remain largely unexplored. In this study, we focus on the type 1 incoherent feed-forward loop (I1-FFL) gene circuit with four different variations (TX, TL, HY-1, HY-2), to investigate how regulation at the transcription and translation levels affect the circuit dynamics. We develop a mechanistic model for each of the four circuits and deploy sensitivity analysis to investigate the circuits’ dynamics in terms of pulse generation. Based on the analysis, we observe that the repression regulation mechanism dominates the characteristics of the pulse as compared to the activation regulation mechanism and find that the I1-FFL with transcription repression has a higher chance of generating a pulse meeting the desired criteria. The experimental results in *Escherichia coli* also confirm our findings from the computational analysis. We expect our findings to facilitate future experimental construction of gene circuits with insights on the selection of appropriate transcription and translation regulation tools.

## Introduction

Synthetic biology is a multidisciplinary field focusing on understanding the underlying networks, dynamics, and mechanisms apart from cellular gene regulation, with the objective to construct synthetic gene circuits that possess functionalities found in natural biological systems as well as novel functionalities.^1,2^ Notable synthetic gene circuits include oscillators,^3,4^ bistable switches,^5,6^ arithmetic circuits,^7^ logic gates,^8^ and biological feedback controllers.^9,10^ Although much of early synthetic gene circuit construction have focused on protein-based regulators, RNA-based genetic circuits have become progressively favored due to their programmability, fast signal propagation, and low cellular burden.^11,12^ Endeavors in RNA-based gene circuits have brought in a variety of tools for both transcription and translation regulations. For example, for transcription regulation, popular tools include small transcriptional activating RNAs (STARs),^13^ which leverage the conditional formation of hairpin for modulating transcription termination via RNA binding, and the CRISPR system, which assists or interferes RNA polymerase recruitment for either transcription activation (CRISPRa) or repression (CRISPRi).^14,15^ Notable tools for achieving translation regulation include ribozyme-based regulators, which utilize sequence sequestration for translation initiation, and toehold switches (THS),^16^ which deploy RNA structural manipulation for either translation initiation or inhibition.

Despite the diverse pool of building tools for gene circuits and the wide range of reported success of utilizing one or more of these tools *in vivo*,^17−19^ it remains largely unexplored how the level of regulation would affect the characteristics of a circuit. Given the increasing complexity of gene circuits, understanding such relationships will tremendously benefit the design and realization of the circuits with predictable dynamics. For example, the repression pathway in the type 1 incoherent feed-forward loop (I1-FFL) circuit can be achieved with a transcription repression via the CRISPRi system,^20^ it can also be realized with a translation repression via a toehold repressor.^21^ An understanding of the similarities and differences in these two different regulation mechanisms would significantly contribute to the design and tuning of the circuit in experiments. Therefore, we explore the impact of different regulation mechanisms on the characteristics of the I1-FFL gene circuit with model-based analysis, with the intention to establish guidelines for regulation tool selection for future gene circuit construction.

The I1-FFL circuit consists of three nodes (i.e., X, Y, Z) connected by two regulatory pathways that operate in opposition, where X activates gene Y and the target gene Z, while gene Y represses Z. Due to this incoherence, the I1-FFL circuit can generate a pulse in the target gene and has attracted numerous interests due to its wide applicability as a response accelerator,^22^ bandpass filters,^23,24^ fold change detection,^25,26^ biosensing,^27^ and noise buffering.^28,29^ The I1-FFL circuit is one of the simplest and most studied gene circuits, and the circuit structure also contains both activation and repression pathways that can be achieved with different RNA-based regulation tools. These topological features render the I1-FFL an excellent model for our study.

Built on physical understanding and certain assumptions, mechanistic models aim to capture the dynamics of gene regulatory networks by representing the molecular-level interactions as chemical reactions. In addition to describing circuit dynamics, a mechanistic model can provide details regarding the individual component, thus enabling an in-depth investigation of the relationship between the constituent components and the overall dynamics of the circuit. These mechanistic models typically composed of ordinary differential equations (ODEs) are a powerful tool for predicting performance and fine-tuning circuit design. In our previous work, we have demonstrated the effectiveness of using mechanistic models to predict RNA-based I1-FFL circuit dynamics by integrating experimental data for the model parameterization.^30^ Here, we perform our analysis based on ODE models that feature a general mechanistic description of the chemical reactions to establish a universal model for each circuit that can be easily modified for experimental implementation.

In this work, we started by designing four I1-FFL circuit variations based on regulation pathways at either the transcription or the translation level. We then developed mechanistic models for each of the circuits and defined performance metrics to quantify our computational analysis. With local sensitivity analysis, we explored the achievable dynamics of each circuit by manipulating a single parameter. The results indicate similar effects from varying individual parameter values for the transcription downregulation circuits (TX and HY-2) and the translation downregulation circuits (TL and HY-1). Using a global sensitivity analysis with a Latin Hypercube Sampling approach, we examined the relationship between the kinetic parameters and the overall circuit performance. Results demonstrate that the transcription downregulation circuits (TX and HY-2) gave the highest number of simulations that met our specified performance criteria, while the two translation downregulation circuits (TL and HY-1) gave a similarly small number of simulations that met our specification. These findings indicate that the repression regulation mechanism of the I1-FFL circuit might dictate the pulse characteristics more than the activation regulation mechanism, and the transcription repression seems to offer a higher flexibility in designing the circuit, as compared to the translation repression. To verify the findings, we then constructed the TX Circuit and evaluated the performance in *E. coli*. Experimental results confirmed the achievability and tunability of a pulse generation in the GFP concentration, which validated the conclusions from our computational analysis. We expect the findings in this study to benefit future gene circuit design not only for I1-FFL but also for other circuits that involve repression and activation regulations.

## Results

### Mechanistic Model of the I1-FFL Circuits

**Figure 1:**
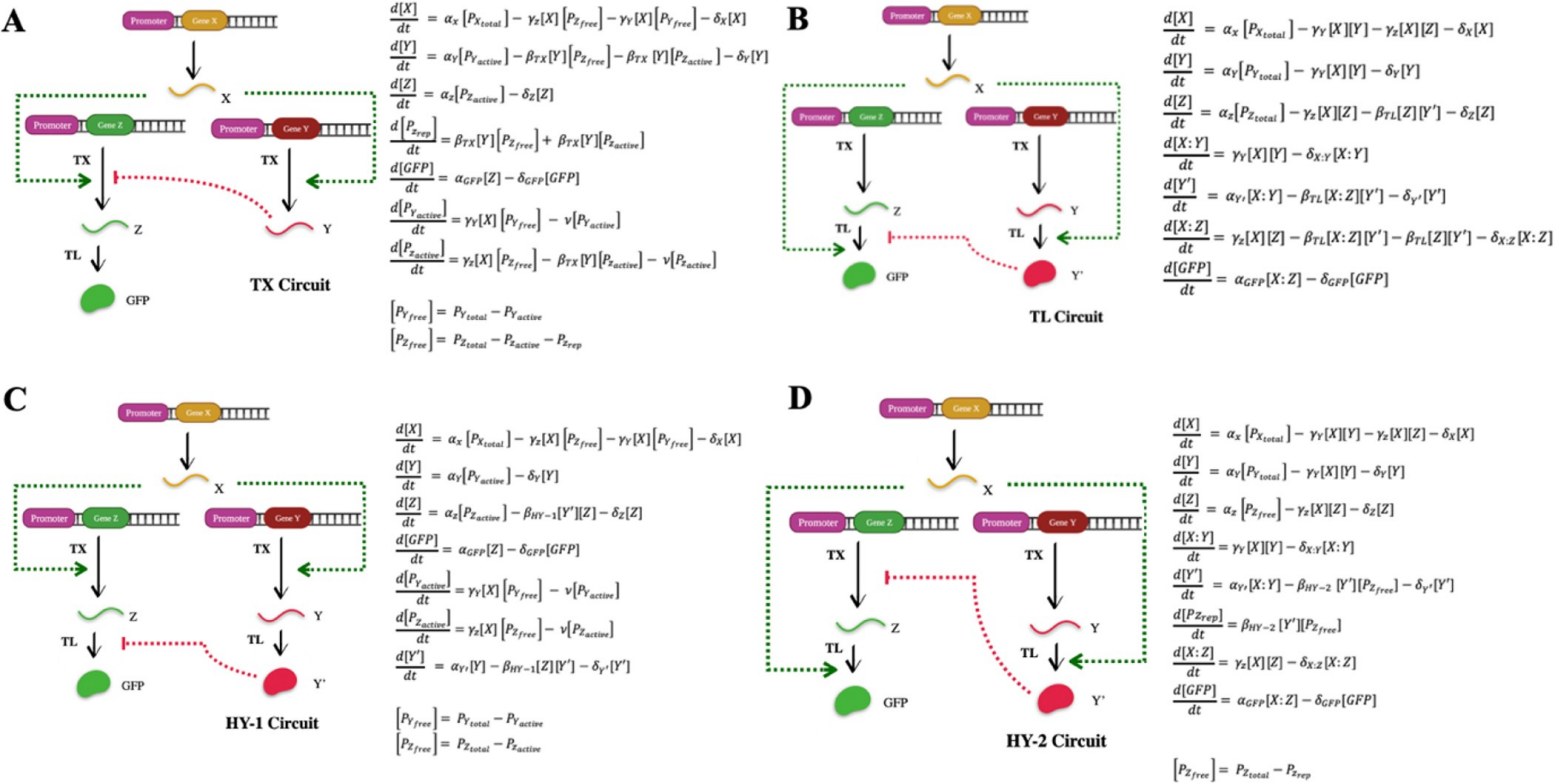
Four designs of the I1-FFL circuits with the corresponding mechanistic model. (A) TX Circuit, featuring transcription regulation for both activation and repression. (B) TL Circuit, featuring translation regulation for both activation and repression. (C) HY-1 Circuit, featuring transcription activation and translation repression. (D) HY-2 Circuit, featuring translation activation and transcription repression.

We first developed simplified mechanistic models for the four I1-FFL genetic circuits, as shown in Figure 1. Specifically, we highlight nucleic acid interactions for transcription and translation regulations and lump detailed dynamics such as RNA polymerase, small molecule inducers, and ribosome activities into related kinetic parameters. Although such a simplified mechanistic model does not encompass the exhaustive dynamics of the system, easy modifications with extra ODEs can expand the model to cover more details for experimental implementation. Note that the focus of this work is to highlight and investigate the relationship between regulation level and the pulse properties of the I1-FFL gene circuit. Therefore, we explore variations of the I1-FFL circuit by alternating the regulation level (transcription or translation) between the two types of regulation pathways (activation or repression) and focus on the four specific designs.

The first I1-FFL circuit is named as TX Circuit (Figure 1A) and is designed to feature regulations only at the transcription level. In this design, the *X* RNA is constitutively transcribed from plasmid *P_X_total__* at rate α_*X*_. The transcription of both *Y* and *Z* RNA is initially off, until *X* RNA binds to the free plasmid *P_Y_free__* or *P_Z_free__* at rate γ_*Y*_ or γ_*Z*_, to form the transcription-active state *P_Y_active__* or *P_Z_active__*. Repressor RNA transcript *Y* can bind to both the free *Z* plasmid *P_Z_free__* and the transcription-active *Z* plasmid *P_Z_active__* at a binding rate of β_*TX*_ to repress the transcription activity. The output GFP is produced at rate α_*GFP*_, and degrades at rate δ_*GFP*_. For experimental realization, we consider the STAR mechanism for the transcription activation and the CRISPRi system for the transcription repression.

The second I1-FFL circuit is named as TL Circuit (Figure 1B) and is designed to feature regulations only at the translation level. In this design, *X* RNA is constitutively transcribed from plasmid *P_X_total__* at rate α_*X*_. However, instead of targeting the downstream plasmids, *X* RNA would target *Y* and *Z* RNA for translation initiation. This mechanism can be realized with THS, a riboregulatory system that regulates the gene expression at the translation level. Specifically, *X* RNA in this case would be the conjugate trigger RNA, which would unwind the hairpin (*Y* and *Z* RNA) through toehold-mediated strand displacement reaction to form complexes *X*:*Y* and *X*:*Z* at rate γ_*Y*_ and γ_*Z*_ , respectively.^31^ Once the THS complex is formed, *X*:*Z* can undergo translation to produce GFP at rate α_*GFP*_. On the other hand, the *X*:*Y* complex is translated to form *Y*^′^, an RNA binding protein or a Cas13a that targets *Z* RNA for translational inhibition at rate β_*TL*_.^32−34^

The third and fourth I1-FFL circuits feature a combination of both the transcription and translation regulations as illustrated in Figure 1C,D. The first type is named as HY-1 Circuit and is designed with transcription activation of *Y* and *Z* by *X* but with a translation repression from *Y* to *Z*. Such a design can be realized by using STAR for the activation, and *Y* being a trigger RNA for the translation repression of *Z* that contains a 3WJ repressor.^21^ The second type is named as HY-2 Circuit and features translation activation of *Y* and *Z* by *X*, with *Y* repressing the GFP output at the transcription level. In this scenario, *Y* and *Z* translation activation can be achieved with the THS mechanism as described in the TL Circuit, and the transcription repression of *Z* can be achieved with the CRISPRi system as described in the TX Circuit.

### Performance Metrics for Local and Global Sensitivity Analysis

**Figure 2:**
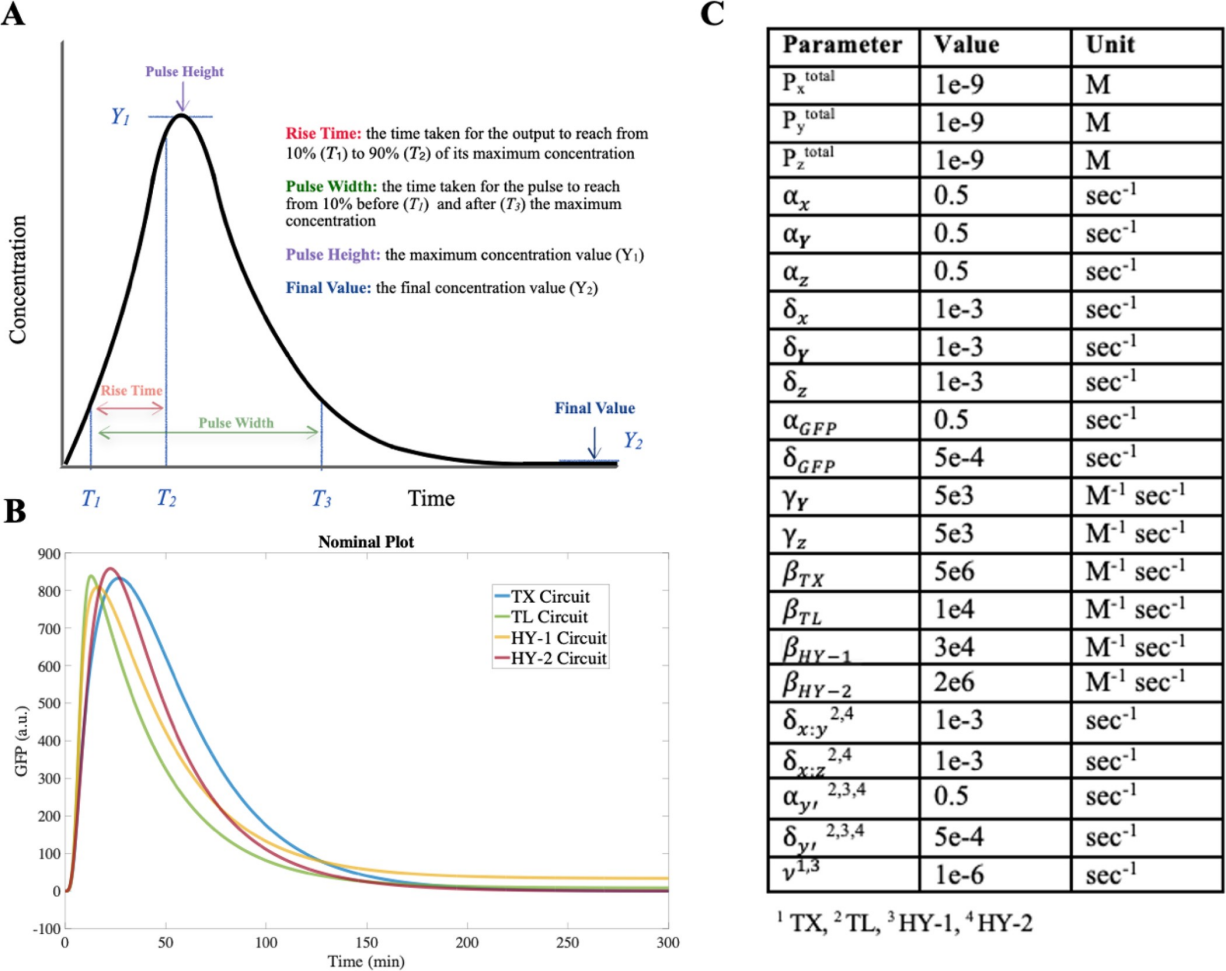
(A) Pulse metric definitions: rise time, pulse width, pulse height, and final value. (B) Nominal simulation plot with the corresponding parameter value. (C) Tables of nominal parameter values.

Referring to previous studies on the I1-FFL circuits^35,36^ and standard concepts from systems and control theories,^37^ we define four performance metrics (Figure 2) to quantify the dynamics of the Z output as follows: rise time (TR) defined as the time taken for the output to reach from 10 (*T*_1_) to 90% (*T*_2_) of its maximum concentration; pulse width (PW) defined as the time taken for the pulse to reach from 10% before (*T*_1_) and after (*T*_3_) the maximum concentration; pulse height (PH) defined as the maximum concentration value (*Y*_1_); and final value (FV) defined as the final concentration value (*Y*_2_). These metrics provide crucial context regarding the performance of the circuits for the local and global sensitivity analysis. We further impose quantitative requirements on these metrics to ensure that each simulation produces a characteristic pulse. The four requirements are defined as follows: maximum output must occur before a cutoff time point (5 h), FV ≤ 10% of maximum output value, TR ≤ 150 min, and PW ≥ 30 min. These criteria are selected based on heuristic experience for a decent response speed (rise time) to changes, a reasonable pulse duration (pulse width) and settling time, and also on our previous computational study on a biological feedback controller, which showed the suitability of these criteria in analyzing circuit response to a step change.^38^

Since our analysis relies on the characteristics of the output GFP concentration profile, it is critical to have each of the circuits start with a similar GFP profile. By tuning the kinetic parameters, we identified a common set of biologically relevant kinetic parameters, except for β, the binding rate between the repressive node, *Y*, and the output *Z*. The nominal parameter set that makes all four circuits produce a similar GFP concentration profile is shown in Figure 2. The mathematical models for each of the four circuits are described in Figure 1 where α is the production rate, γ and β are the binding rates, and δ is the degradation rate. *P*_X *total*_, *P*_Y *total*_, and *P*_Z *total*_ are the total plasmid concentrations for *X*, *Y*, and *Z*, respectively.

### Local Sensitivity Analysis: Investigating the Impact of Single Parameter Manipulation

With the proposed designs, we first investigate what dynamics each of the four circuits could achieve by manipulating the model parameters. For this purpose, we performed a local sensitivity analysis, where a single parameter is varied within a specified range in each simulation, and the performance is then analyzed using the four performance metrics. This analysis investigates the effect of changing only one parameter at a time on the output dynamics and could provide guidelines for component selection for experimental realization. For example, the transcription rate can be changed with different promoters, and the translation initiation rate can be changed with different ribosome binding sites. For each simulation, we varied one parameter by multiplying with a “multiplication factor”, which ranges from 10^–3^ to 10^3^ on a logarithmic basis. Depending on the number of model parameters, we conducted a total of 660, 825, 770, and 825 simulations for the TX, TL, HY-1, and HY-2 Circuits, respectively. With the requirements specified in the previous section, we found that all four circuits have yielded a similar percentage of simulations that met our specification, despite slight differences in the TL and HY-2 Circuits. Specifically, we obtained 70, 65.8, 70.9, and 75.5% for the TX, TL, HY-1, and HY-2 Circuits, respectively.

**Figure 3:**
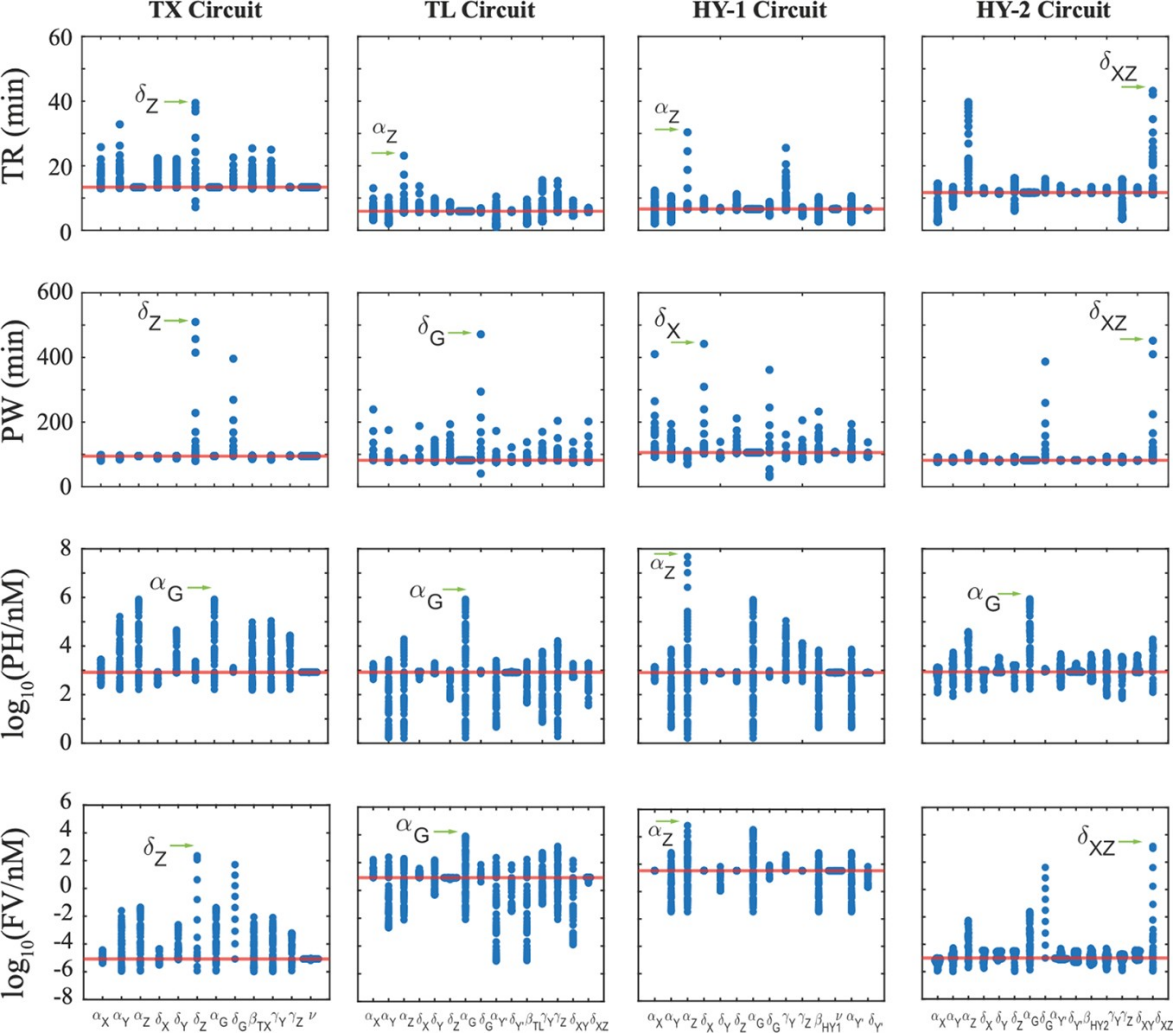
Overall distribution of the metric across all four circuits. Red line represents the metric value at nominal parameter values. Blue data points represent the simulated metric values subject to each parameter variation. The most sensitive parameters for each metric across all four circuits are shown with green arrows for better visualization.

The local sensitivity analysis results in Figure 3 reveal several key observations about the tunability of the dynamics (see also Figures S1–S4). In general, individually perturbing the kinetic parameters shows a more prominent effect on elongating the rise time (TR) and expanding the pulse width (PW), with a tunable range of 0.2× to 4× for the rise time and 0.5× to 4× for the pulse width obtained with the nominal values (indicated by the red horizontal lines). Interestingly, the transcription downregulation circuits (TX and HY-2) demonstrate a similar response to individual kinetic parameter manipulation, in which such a single parameter manipulation tends to have a higher chance of increasing the pulse height (PH) and the final value (FV), instead of decreasing the two metrics. On the contrary, single parameter manipulation in the translation downregulation circuits (TL and HY-1) shows both a moderate increase and decrease in the two metrics. This observation further suggests that different types of regulation might lead to distinct dynamics, and the repression pathway might dominate such effects as compared to the activation pathway in the I1-FFL circuit.

After investigating the range of the dynamics each circuit can achieve, we proceeded to identify parameters that most significantly affect the circuit dynamics. Such an understanding could enable us to adjust the circuit dynamics most effectively by tweaking a small number of parameters, thus reducing experimental costs for component design. The first observation we note in Figure 3, based on the corresponding achievable maximum and minimum values, is that the parameters that have the most significant impact are those associated with either the *Z* mRNA (α_*Z*_, δ_*Z*_) or the GFP (α_*G*_, δ_*G*_). This is expected since these parameters would directly determine the GFP concentration. We also note that besides the four *Z* mRNA and GFP-related parameters (α_*Z*_, δ_*Z*_, α_*G*_, and δ_*G*_), there are several other parameters that could also contribute to similar relative fold-changes across all four circuits, such as α_*X*_ and α_*Y*_.

**Figure 4:**
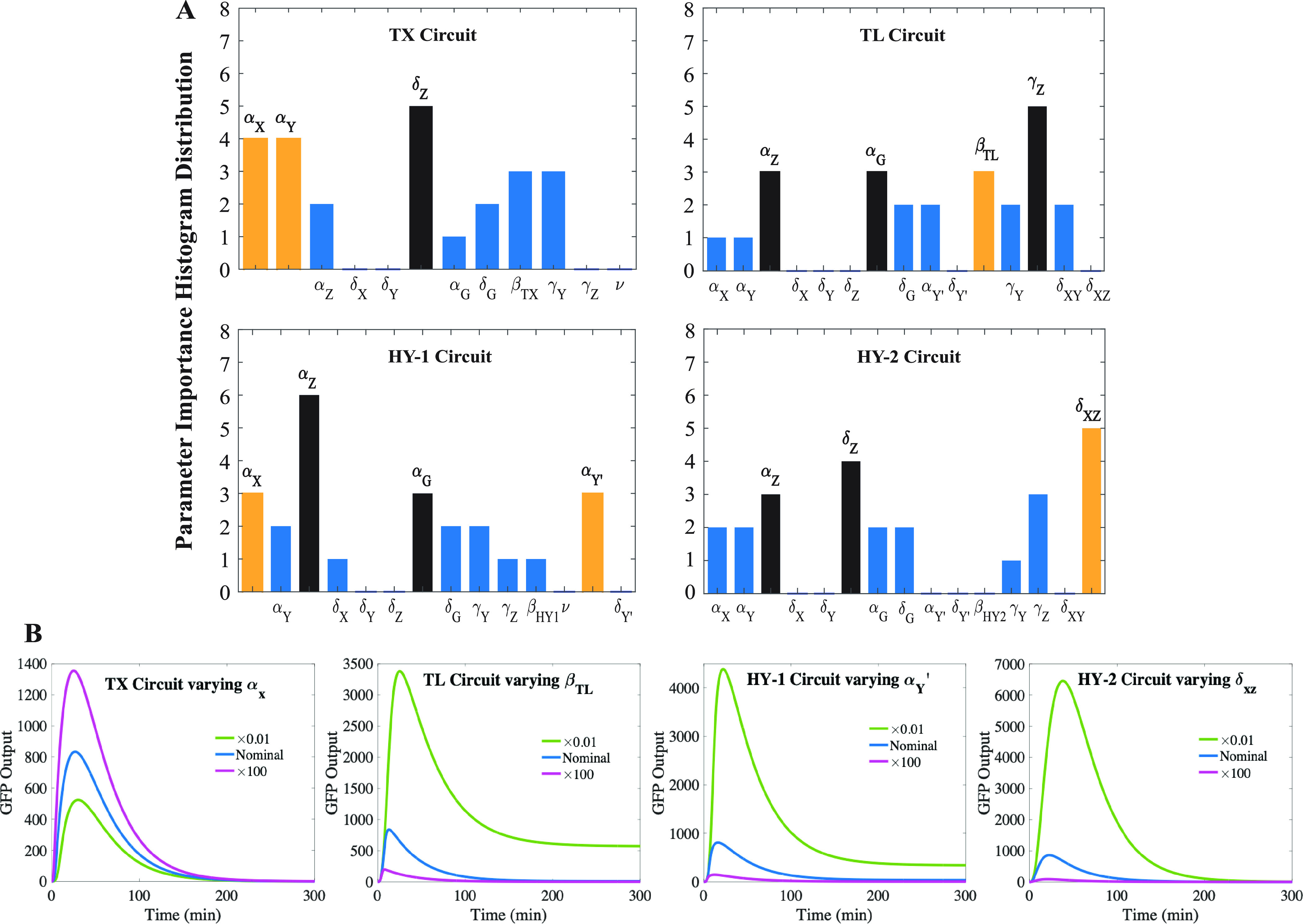
Local sensitivity analysis results: (A) Parameter importance histogram distribution showing the most impactful parameters (colored in either black or yellow) in affecting the maximum and/or minimum value of the four performance metrics. The black bar indicates parameters related to the Z component and GFP, and the yellow bar represents the most impactful non-Z component or GFP-related parameters. (B) Individual simulations for each circuit show the effects of varying the most impactful non-Z component or GFP-related parameters.

To facilitate the analysis, we focus on the top three parameters that most significantly affect the maximum value of each metric and the top three parameters that most significantly affect the minimum value of each metric. Therefore, each parameter could potentially be found impactful in eight scenarios (four metrics and two directions, maximum and minimum) for each circuit. Given that the nominal values for each circuit are slightly different, to ensure a fair comparison, we analyze the relative fold-change over the nominal value. We then summarize the total number of times that each parameter is found as the top three most impactful parameters in Figure 4, for a quantitative analysis. The histogram is interpreted as the following: for example, δ_*Z*_ is found five times in the TX Circuit, meaning that it is identified as one of the top three impactful parameters in five out of the eight scenarios for the TX Circuit, across all four metrics. Reading off the histogram, we can then identify the most impactful parameters for each circuit. Excluding the four *Z* mRNA and GFP-related parameters, we found that the most impactful parameters for the TX Circuit are α_*Y*_ and α_*X*_; that for the TL Circuit is β_*TL*_; that for the HY-1 Circuit are α_*X*_ and α_*Y*^′^_; and that for the HY-2 Circuit is δ_*XZ*_.

We also observe that the transcription downregulation circuits (TX and HY-2) share the common impactful parameters α_*X*_ and α_*Y*_, while the translation downregulation circuits (TL and HY-1) share the common parameters α_*Y*^′^_ and γ_*Y*_. This again suggests that circuits with the same repression pathway tend to show a higher similarity in their properties. The complete results are provided in Figure S5.

### Global Sensitivity Analysis: Investigating the Designability of Each Circuit

The local sensitivity analysis provides insight into the effect of perturbing a single parameter on the output dynamics of each circuit. To complement the analysis, we also performed a global sensitivity analysis for a holistic understanding of how the kinetic parameters would affect the dynamics of the four models cooperatively. This type of analysis is accomplished by simultaneously varying all the parameters randomly in each simulation, and the performance of each circuit is analyzed in terms of the same four metrics as used in the local sensitivity analysis.

To ensure an unbiased sampling of parameter values, we adopted the Latin Hypercube Sampling^39,40^ approach to randomly generate a parameter that is within 10^–2^ to 10^2^ of the nominal value of each parameter. In the Latin Hypercube Sampling, each parameter is discretized into *n* evenly spaced intervals (corresponding to a total of *n* samples), and each interval is sampled exactly once in the simulation. This approach avoids biased sampling and provides an exhaustive selection of parameter values within the range of interest. For this analysis, each parameter is evenly discretized into 10,000 intervals, generating a total of 10,000 random combinations of parameter sets for each of the four circuits. Same as in the local sensitivity analysis, only simulations that met the performance criteria are analyzed.

**Figure 5:**
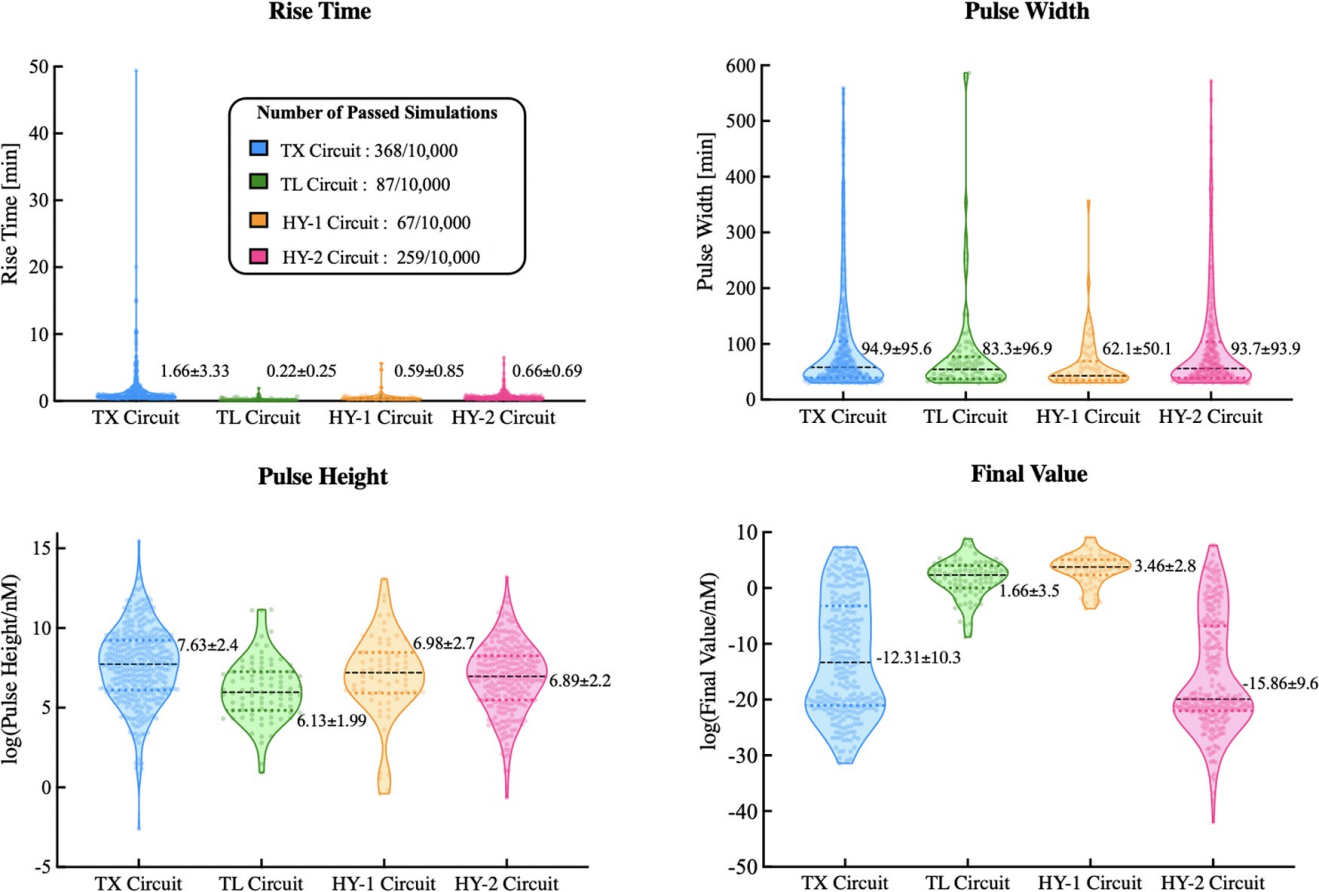
Global sensitivity analysis showing the achievable dynamics of each circuit: Violin plot distribution of rise time, pulse width, pulse height, and final value across all four circuits. Each data point represents the parameter value of a specific simulation; the shaded regions are the kernel density estimated; and the dashed lines correspond to the mean and quartile range of each distribution. In addition, each violin plot is annotated with the numerical values corresponding to the mean and standard deviation.

According to the results in Figure 5, we found that out of the 10,000 simulations, the TX and HY-2 Circuits gave the highest number of simulations that met our criteria (368 and 259 simulations specifically), while the TL and HY-1 Circuits had the lowest number of simulations with 87 and 68, respectively. This observation suggests that a transcription downregulation might provide a higher flexibility in parameter design to achieve a desirable pulse response, in comparison to a translation downregulation.

A closer inspection of results in Figure 5 reveals several findings. First, the rise time distribution is similar across all circuits except that the TX Circuit shows simulations with significantly higher rise time. While most of the simulations produced a pulse width between 30 and 100 min in all four circuits, the TX and HY-2 Circuits had more simulations with a pulse width above 100 min. This observation suggests that for a longer pulse duration, the TX and HY-2 Circuits might be a more suitable choice. The pulse height distribution also shows a high similarity across all four circuits, with the exception that the TL Circuit produced on average a lower pulse height, as compared to the other three. The most prominent difference is observed in the final value distribution, where the transcription downregulation circuits (TX and HY-2) have lower final values than the translation downregulation circuits (TL and HY-1). Furthermore, the final value distribution indicates that the TX and HY-2 Circuits have a higher chance of adapting to the initial concentration after the pulse, as compared to the TL and HY-1 Circuits, given the fact that the output concentration in all simulations was initiated at zero. Note that the *y*-axis for the pulse height and final value is in natural log.

**Figure 6:**
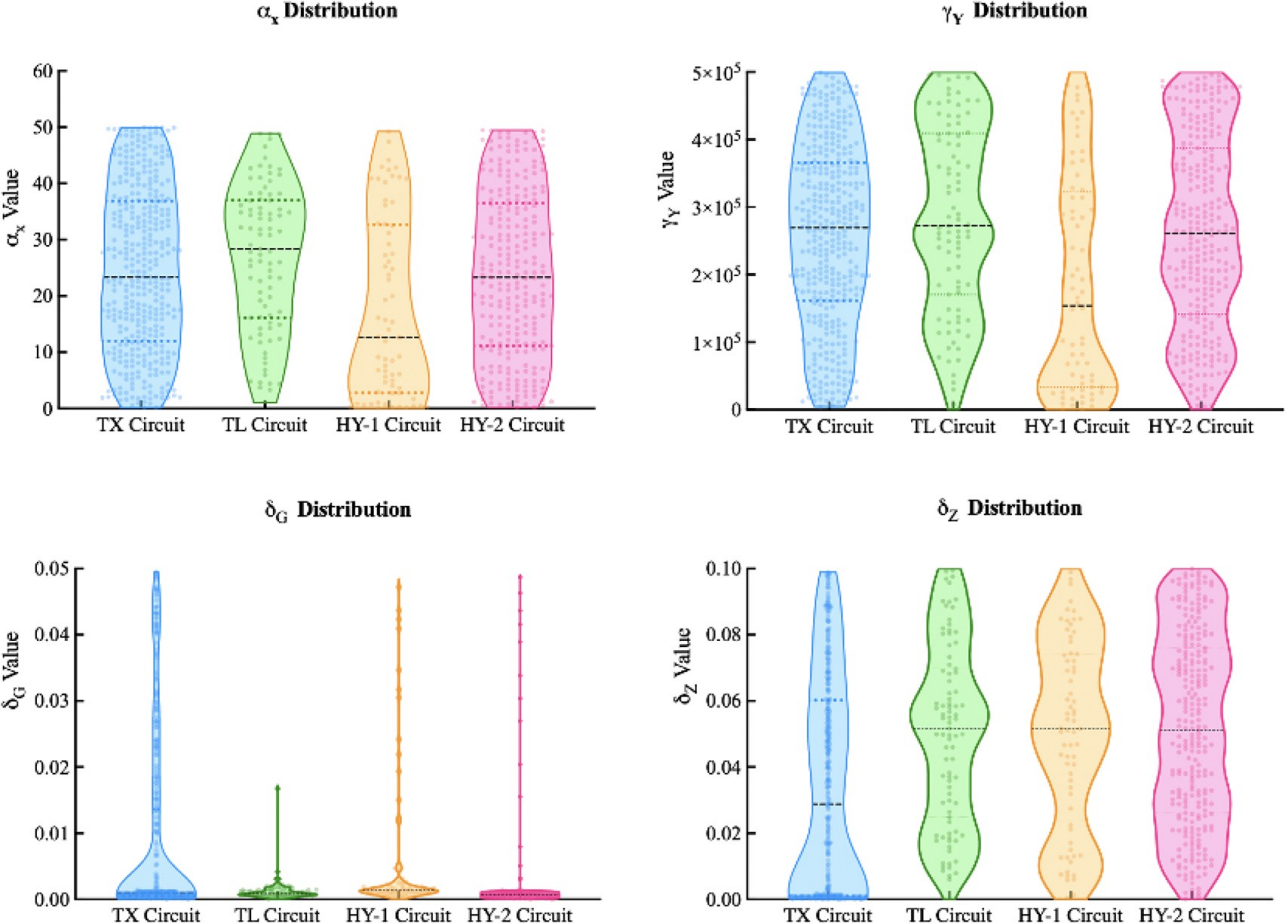
Global sensitivity analysis showing the required kinetic parameter values for each circuit to achieve the desired dynamics: Violin plot distribution of α_*X*_, γ_*Y*_, δ_*G*_, and δ_*Z*_ across all four circuits. Each data point represents the parameter value of a specific simulation; the shaded regions are the kernel density estimate; and the dashed lines correspond to the mean and quartile range of each distribution.

Figure 5 reveals the similarities and differences among the four circuits, in terms of potentially achievable dynamics (in terms of the four metrics), and the easiness of achieving specific dynamics (the number of passed simulations), by randomly perturbing all the design kinetic parameters. To complement the comparison, we then proceeded to investigate the similarities and differences in terms of the required parameter values for each circuit to achieve the desired dynamics. To highlight the key observations from our results, we have summarized the parameter value distribution of four specific parameters in Figure 6. Other parameter value distributions are provided in the Figures S6 and S7. The distributions of parameters α_*X*_ and γ_*Y*_ indicate that both the TX and the HY-2 Circuits have a more even distribution than the TL and HY-1 Circuits. This means that the specified pulse property can be achieved with any parameter in the specified range with a similar probability. However, a higher α_*X*_ value would have a higher chance of obtaining the specified pulse property for the TL circuit, while a lower α_*X*_ value would be favored in the HY-1 Circuit. We also note similar observations in the γ_*Y*_ distribution. The δ_*G*_ distribution indicates that a low GFP degradation rate would be needed for all four circuits to produce a pulse that meets our specification. The δ_*Z*_ distribution of the TX Circuit shows a prominent bimodal profile and shows a slightly bimodal profile for the HY-2 Circuit, indicating a higher chance of obtaining the specified property with parameter values from either the top or the bottom region of the specified parameter range. These observations again suggest a higher similarity between circuits with the same downregulation mechanism (TX vs HY-2 and TL vs HY-1).

### Experimental Construction of the TX Circuit

In our previous work, Hong et al.,^30^ we obtained a pulse generation with an HY-2 Circuit but not the HY-1 Circuit; this finding agrees with the simulation analysis presented here, which suggests that the TX and HY-2 Circuits have the highest chance of generating a pulse. Therefore, we then sought to perform a quick experimental validation on whether the TX Circuit can generate a pulse or not, with moderate efforts.

**Figure 7:**
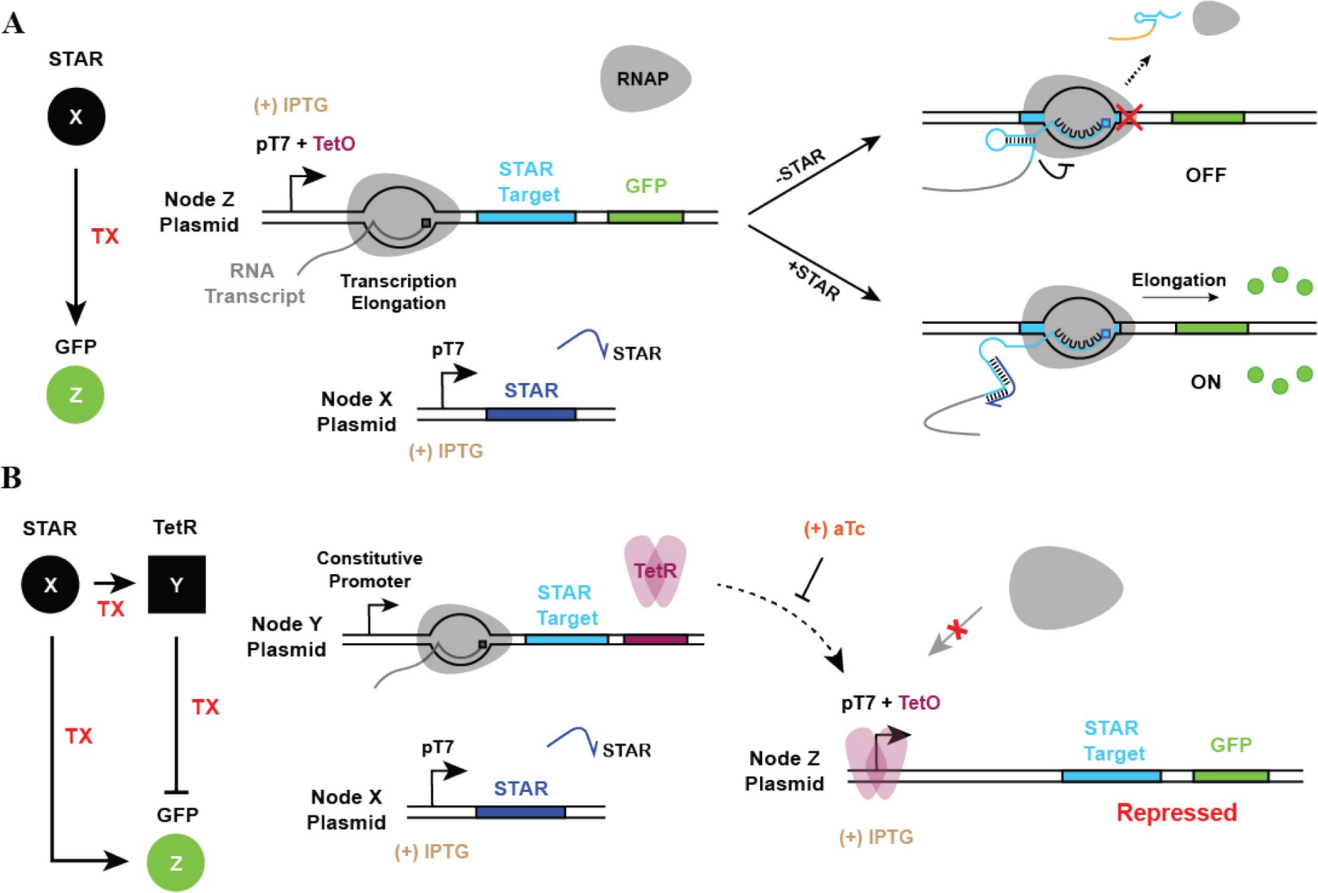
The TX Circuit is composed of the STAR activation and the TetR repression pathway. (A) Schematic of the *X* to *Z* activation pathway. Binding of *X* to the STAR target of *Z* prevents terminator formation to allow transcription elongation. (B) Schematic of the comprehensive TX I1-FFL circuit. STAR (*X*) activates the expression of TetR, which binds to TetO to block RNAP access for *Z* transcription repression. aTc treatment is used to release TetR from TetO within pT7 to restore GFP expression.

The experimental construction of the TX Circuit is realized with STAR being the *X* node to activate the transcription of nodes *Y* and *Z*, whereas node *Y* (once activated) encodes a TetR protein for transcription repression of node *Z*, as shown in Figure 7. Specifically, the *X* node contains a STAR under the control of pT7, the *Y* node contains the STAR target followed by TetR under the constitutive promoter, and the output node *Z* contains a STAR target followed by a GFP reporter under the control of pT7, as well as a Tet operator site (TetO) for transcription repression. All the three nodes are encoded in separate plasmids to allow different combinations of circuit components to be tested in *E. coli*. The detailed protocol of the plasmid construction is provided in Methods. These plasmids are then transformed in *E. coli* BL21 DE3 carrying genomic T7 RNAP under the control of the Lac promoter such that the expression of *X* and *Z* can be controlled by the concentration of IPTG, while the expression of *Y* is constitutive. The regulatory strength of node *Y* can also be tuned, by either adjusting the promoter strength of *Y* or by adding a small molecule inducer anhydrotetracycline (aTc) that titrates TetR. Therefore, we constructed the *Y* node with a strong promoter J23119 and a weak promoter J23110 for the STAR target expression and tested the performance of the circuits with different concentrations of aTc.

**Figure 8:**
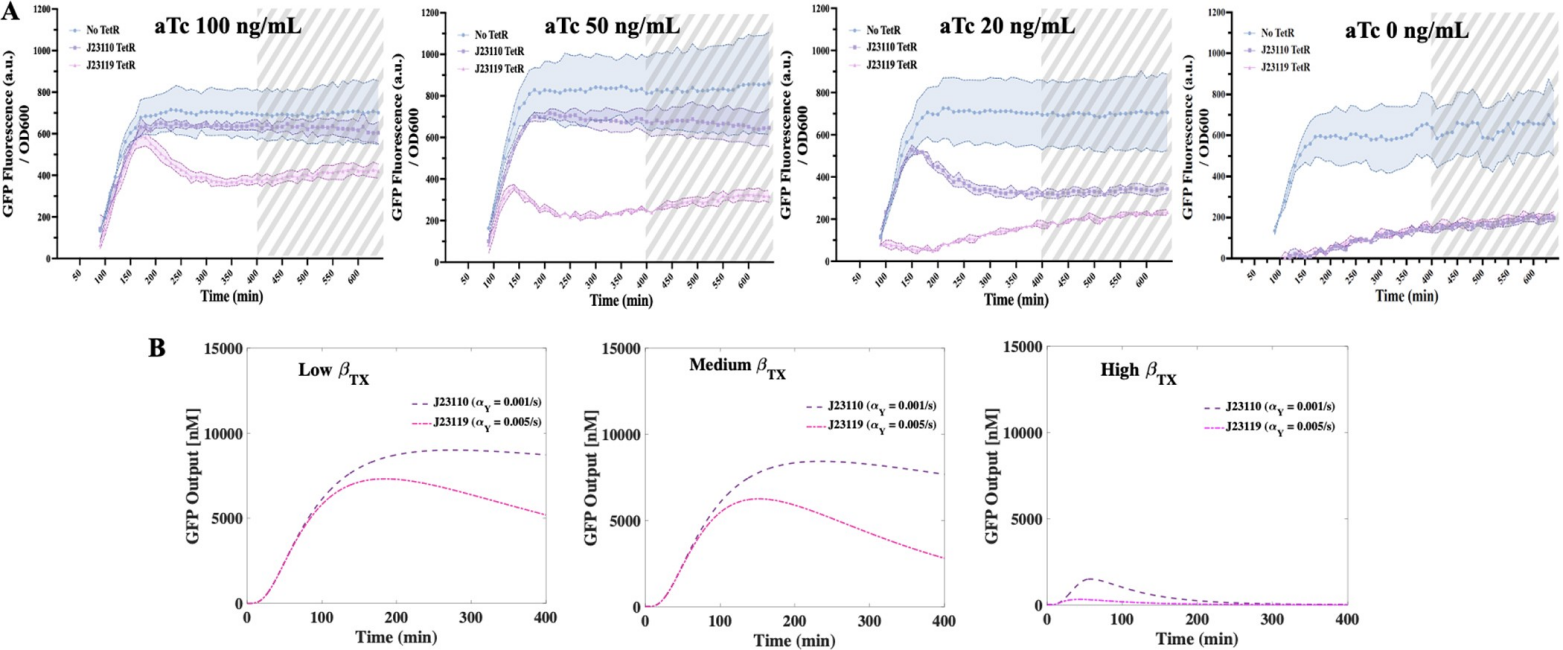
Experimental and computational visualization of TX Circuit dynamics. (A) Experimental validation of the TX Circuit confirms the achievability and tunability of a pulse in the output GFP concentration. Time course of GFP fluorescence measurements with inducer IPTG concentration of 0.1 mM and aTc concentrations of 100, 50, 20, and 0 ng/mL. Data for the first 80 min are removed due to the low OD600 values, and the time points beyond 400 min are marked as gray dashed areas to indicate the transition to the stationary phase. For the metric quantification of these plots, see Table S4. (B) Simulation plots capture qualitatively the behavior observed in experiments by varying the related kinetic parameters. Three levels of β_*TX*_ along with two levels of α_*Y*_ are screened to mirror the inducer concentration and promoter strength variables investigated in the experiments.

Figure 8A summarizes the experimental GFP readout on the TX Circuit in *E. coli*, subject to four different aTc concentrations (100, 50, 20, and 0 ng/mL), with 0.1 mM of IPTG. Several observations we notice are as follows: (1) by tuning the promoter strength and the aTc concentration, we obtained prominent pulse generation in both the TX Circuits, see results for aTc = 20, 50, and 100 ng/mL. This aligns with our simulation findings that tuning α_*Y*_ (i.e., promoter strength) could effectively manipulate the dynamics, as α_*Y*_ being found one of the most impactful kinetic parameters for the TX Circuit. (2) To achieve a pulse generation, a higher aTc concentration will be needed to compensate the enhanced transcription of a stronger *Y* promoter. Note that for aTc = 50 and 100 ng/mL, the pulse is only observed for circuit with promoter J23119, whereas for aTc = 0 ng/mL, no pulse is observed for both promoters. The detailed characterization of the pulse using the four performance metrics are given in the Supporting Information, Table S4. To crosscheck the dynamics, we identified the most closely related kinetic parameters in the model to the two variables in the experiments, promoter strength and inducer concentration, as α_*Y*_ and β_*TX*_, respectively, and then performed simulations under various conditions as in Figure 8B. Specifically, the three levels of β_*TX*_, low, medium, and high, are used to simulate the experiments with high, medium, and low/zero aTc concentrations, while the two levels of *Y* transcription rate α_*Y*_ are used to simulate the dynamics with the two promoters in experiments. Although we do not expect our model to capture the details of the experiments as it was developed for analysis at the higher level, the simulations in Figure 8B do agree qualitatively with our experiments that a higher aTc concentration (i.e., lower β_*TX*_ value) leads to an earlier turning point with a lower expression level in the GFP concentration, and as the aTc concentration approaches 0 ng/mL, it becomes harder to achieve a pulse generation. Note that simulation for aTc = 0 ng/mL would require β_*TX*_ to approach infinity, which is not biologically feasible. Therefore, we adopted a large yet biologically feasible value of β_*TX*_ to simulate scenarios where aTc concentration approaches zero, and this resulted in the small pulse in the simulation (High β_*TX*_). In summary, we confirmed that the TX Circuit can exhibit a pulse generation *in vivo* by tuning the key parameters identified from our computational analysis.

## Discussion

Advancements in RNA synthetic biological techniques have brought in a rich library of regulatory tools for the construction of RNA gene circuits at both the transcription and translation regulation levels. However, there are few studies that examine the effect of regulation mechanism on the circuit dynamics. In this work, we leverage the I1-FFL circuit topology to inspect how transcription and translation regulations would affect the pulse generation capability of I1-FFL circuits and the property of the pulse generated. Both the local and global sensitivity analysis indicate higher similarities between circuits with the same downregulation mechanism and provide evidence suggesting that the downregulation pathway might be more critical than the activation pathway of the I1-FFL circuit in determining the circuit property. Moreover, the global sensitivity analysis suggests that an I1-FFL circuit with transcription repression might have a higher chance of achieving a pulse with preferred properties, as indicated by the higher number of successful simulations. This observation agrees with our experimental results on the TX Circuit presented in this study and the experimental results on HY-1 and HY-2 Circuits in the previous study.^30^

The mechanistic models developed in this study have represented the detailed dynamics and interactions in the system in lumped kinetic parameters, with the parameter values inferred from previous experiments. While the model is able to predict the circuit dynamics in experiments, as exemplified by the *E. coli* experiments presented here, we anticipate a detailed model that accounts for the specific experimental designs that would further improve the prediction accuracy, as demonstrated in our previous work on the HY-1 and HY-2 Circuits.^30^ For future studies, we will pursue experimental realization of a broader design of the I1-FFL circuits and with models tailored for each specific design for a more comprehensive examination on how regulations at different gene expression levels would affect the circuit dynamics. We will also expand our study to incorporate other circuits such as oscillators and toggle switches, where timescale is critical, to further analyze the effects of translation and transcription regulation in determining the dynamics of the gene circuits.

The work presented here demonstrates the capabilities of using mechanistic modeling to understand the relationship between the regulation level and the I1-FFL circuit dynamics. As the number of available regulatory components continues to evolve, we anticipate a growing need for mathematical modeling to guide the experimental construction of *de novo* circuits. We anticipate our modeling work to guide the troubleshooting in experiments, for example, the failure in achieving a pulse generation with an RNA-only I1-FFL HY-1 circuit in the previous work.^30^ By providing the crucial information regarding the importance of regulation level in determining circuit performance, we expect our findings, together with the characterization of the subunit gene regulatory parts, to further facilitate the design of synthetic gene circuits with increased complexity and functionality. Resource competition is known to be one of the culprits that lead to the failure of a gene circuit in *in vivo* implementation.^41−43^ Recently, Darlington et al.^44^ proposed a transcriptional and translational-coupled approach to control resource re-allocation that mitigates the effects of resource competition on the performance of gene circuits. Meanwhile, circuit topology has also been found to play a critical role in determining the circuit behavior, especially in response to the effect of cell growth.^45^ Leveraging the topology of the gene regulatory network and using cell growth as feedback, Goetz et al. achieved effective noise control with regulations at both the transcription and translation levels to combat the effect of resource competition.^46^ We envision our findings on how the regulation type (transcription vs translation) would affect the overall circuit dynamics to complete such efforts and to facilitate the design of gene circuits with improved robustness to resource competition.

## Methods

### Model Development and Computational Analysis

The mechanistic models in this study are developed around the key molecular-level interactions in each circuit, following the law of mass action. All the ODEs are solved with MATLAB ode23s for simulations in the local and global sensitivity analysis. The local sensitivity analysis is carried out by varying one of the model kinetic parameters at a time. Specifically, for each simulation, one kinetic parameter for each circuit was multiplied to a “multiplication factor”, which ranges from 10^–3^ to 10^3^ on a logarithmic basis. With the total numbers of model parameters for TX, TL, HY-1, and HY-2 Circuits being 12, 15, 14, and 15, we conducted totals of 660, 825, 770, and 825 simulations for each of the four circuits. Out of these simulations, only those that meet the prespecified metrics of maximum output must occur before 5 h; the final value must be ≤10% of maximum output value; the rise time must be ≤150 min; and a pulse width of ≥30 min is retained for further analysis. For the global sensitivity analysis, all of the parameters for a given mechanistic model are randomly perturbed for each simulation. To ensure unbiased sampling, the Latin Hypercube Sampling Approach was used to generate a parameter between the ×10^–2^ and ×10^2^ range of each parameter’s nominal value. A total of 10,000 simulations were performed for each circuit. Simulations that meet the defined quantitative requirements as in the local sensitivity analysis are analyzed. The violin plots shown in Figures 5 and 6 are plotted using the MATLAB function *violinplot.m* developed by B. Bechtold, which can be downloaded from https://github.com/bastibe/Violinplot-Matlab. All the MATLAB scripts used for simulation in this study can be found at https://github.com/mathiasfoo/4ifflcircuits.

### Plasmid Construction and *E. coli* Strains Used

Plasmids were constructed using PCR, Gibson assembly, and round-the-horn site-directed mutagenesis. All DNA templates for the TX Circuit were assembled from single-stranded DNAs purchased from Bionics. The STAR-target pair sequence was Target Variant 1-STAR Variant 1.^47^ The synthetic DNA strands were amplified via PCR to form double-stranded DNAs. The resulting DNAs were then inserted into plasmid backbones using about 30 bp homology domains via the Gibson assembly.^48^ Promoter change from pT7 to other promoters (J23110, J23119, pT7 (TetO)) was done by round-the-horn site-directed mutagenesis. All plasmids were cloned in the *E. coli* DH5α strain and validated through DNA sequencing. Backbones for the plasmids were taken from the commercial vectors pET15b, pCDFDuet, and pCOLADuet (EMD Millipore). Node X was constructed in pET15b. Node Y and node Z were constructed in pCDFDuet and pCOLADuet. GFPmut3b-ASV was used as the reporter. This GFP is GFPmut3b with an ASV degradation tag.^49^ TetR was used with ASV degradation tag as well. Plasmids were purified using the Enzynomics EZ-Pure Plasmid Prep Kit. Sequences of elements commonly used in the plasmids are provided in Table S2. Plasmids were transformed into strains via chemical transformation. *E. coli* BL21 DE3 strain was used for *in vivo* tests of TX Circuit.

### Cell Culture and Microplate Reader Analysis

Transformed cells were cultured on Luria-Bertani (LB) agar plates (1.5% agar), and then single colonies were inoculated into 500 μL of LB liquid medium supplemented with appropriate antibiotics: pCOLADuet (50 μg/mL Kanamycin), pCDFDuet (50 μg/mL Spectinomycin), pET15b and (100 μg/mL Ampicillin). These cells were grown overnight (∼16 h) in 96-well plates with shaking at 800 r.p.m. and 37 °C. For the TX Circuit, overnight cultured cells were diluted 1/100-fold into fresh medium and returned to shaking (800 r.p.m., 37 °C). After 80 min, diluted cells were induced with the appropriate combination of 0.1 mM isopropyl β-d-1-thiogalactopyranoside (IPTG) and anhydrotetracycline (aTc). aTc was treated at four different concentrations: 100, 50, 20, and 0 ng/mL (200, 100, 40, and 0 nM). An aliquot of 200 μL of inducer-treated cells was added per well on a 96-well black plate (SPL Life Sciences, Pocheon, Korea). Plates were incubated at 37 °C for 10 h 30 min (64 cycles) with double-orbital shaking in a Synergy H1 microplate reader (BioTek, Winooski, VT, USA) running Gen5 3.08 software. GFP fluorescence (excitation: 479 nm, emission: 510 nm) and OD600 were measured at 10 min intervals during incubation. GFP fluorescence levels were normalized as follows: GFP fluorescence for LB blank was subtracted, and the resulting value was divided by OD600. Error bars are the standard deviation from three biological replicates.

## Supporting Information Available

Complete local sensitivity analysis results (Figures S1–S5), complete global sensitivity analysis kinetic parameter distribution (Figures S6 and S7), example of simulations that met the specified performance criteria (Figure S8), plasmid information for experiments (Tables S1–S3), and metric quantification of the experiments (Table S4) (PDF)
