# Supplementary material for: Model-Based Investigation of the Relationship between Regulation Level and Pulse Property of I1-FFL Gene Circuits
Source: ACS Synth Biol. 2022 Jun 22;11(7):2417–28. doi: 10.1021/acssynbio.2c00109 (PMC9295143; doi:10.1021/acssynbio.2c00109)
Supplement: Supplementary file 1 — sb2c00109_si_001.pdf [file sb2c00109_si_001.pdf]

SUPPORTING INFORMATION

Model-based Investigation of Relationship Between Regulation Level and Pulse Property of I1-FFL Gene Circuits

Jordan Ryan[†], Seongho Hong[¶], Mathias Foo[‡], Jongmin Kim[¶], and Xun Tang[†,*]

†Cain Department of Chemical Engineering, Louisiana State University, Baton Rouge, LA 70803, United States of America

‡ School of Engineering, University of Warwick, Coventry CV4 7AL, United Kingdom

¶Department of Life Sciences, Pohang University of Science and Technology (POSTECH), Pohang, Gyeongbuk, 37673, South Korea

*Correspondence: xuntang@lsu.edu

Supplementary Figures

TX Circuit

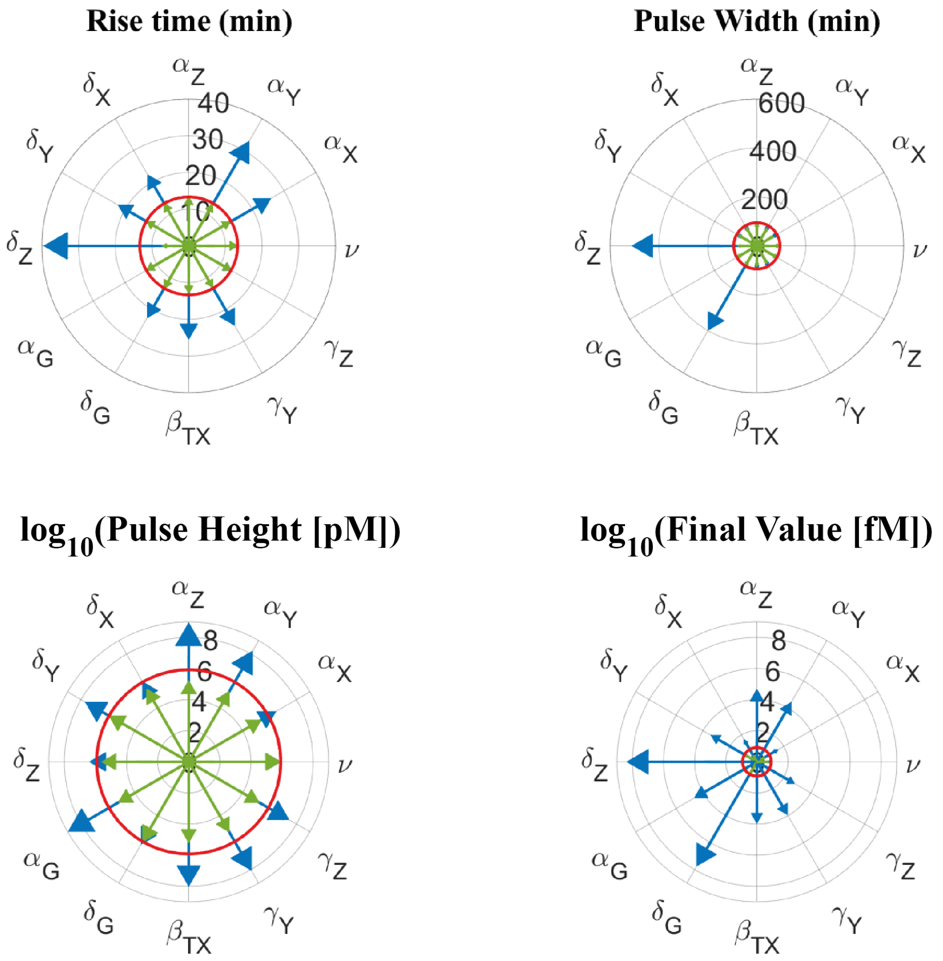

Figure S1. Local sensitivity analysis results for the TX Circuit. The red circle represents the nominal metric value. The blue and green arrows represent the largest and smallest metric values that can be achieved by adjusting the respective parameters, respectively. Using the rise time metric as an illustration, the largest arrowhead and the longest blue arrow for rise time is pointing towards δ_Z indicating that this parameter is the most impactful parameter in realizing the largest rise time. Similarly, the smallest arrowhead and the shortest green arrow for rise time is the δ_Z parameter; therefore, the δ_Z parameter has the highest impact on rise time for the TX Circuit.

TL Circuit

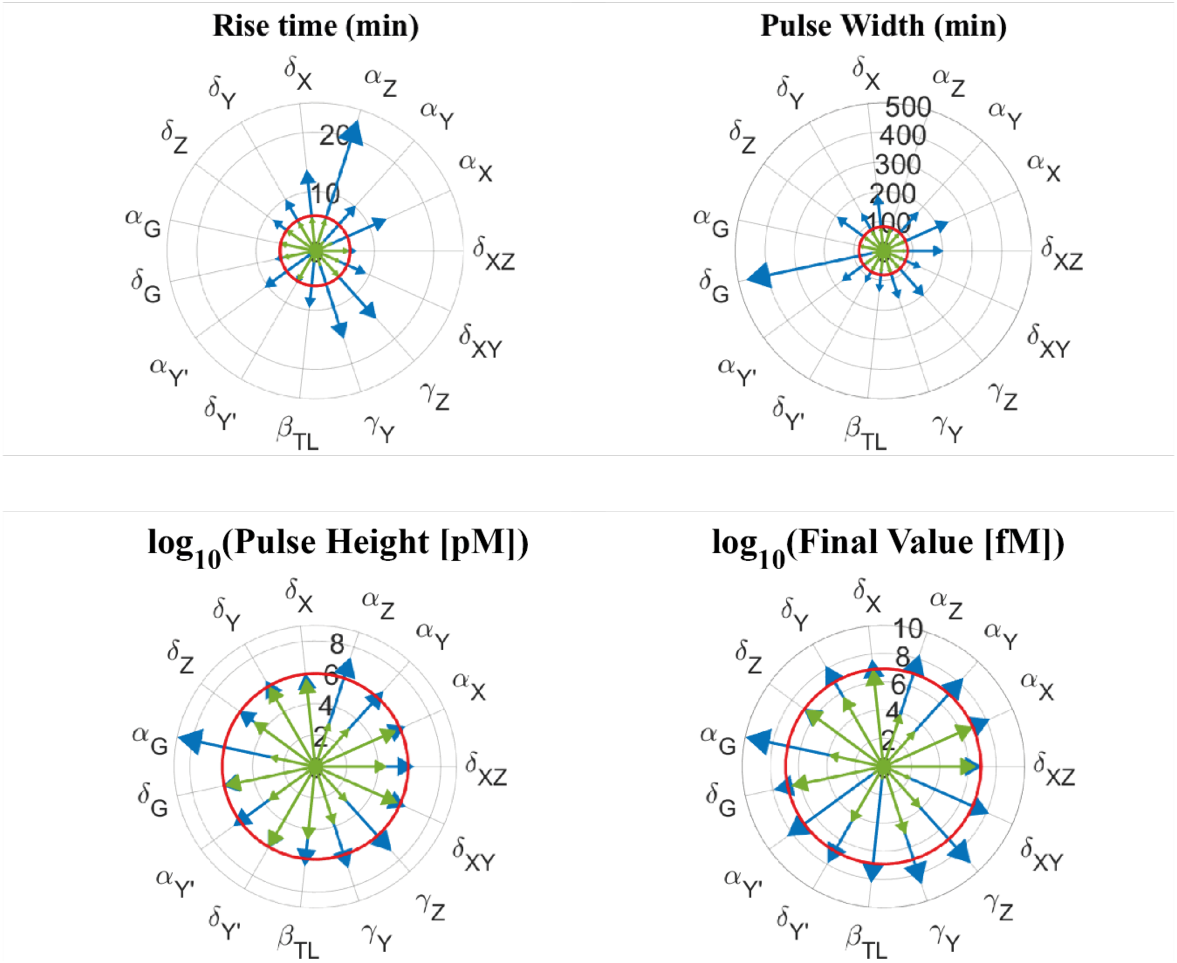

Figure S2. Local sensitivity analysis results for TL Circuit. The red circle represents the nominal metric value. The blue and green arrows, respectively represent the largest and smallest metric values that can be achieved by adjusting the respective parameters. The interpretation follows the description given in the caption of Figure S1.

HY-1 Circuit

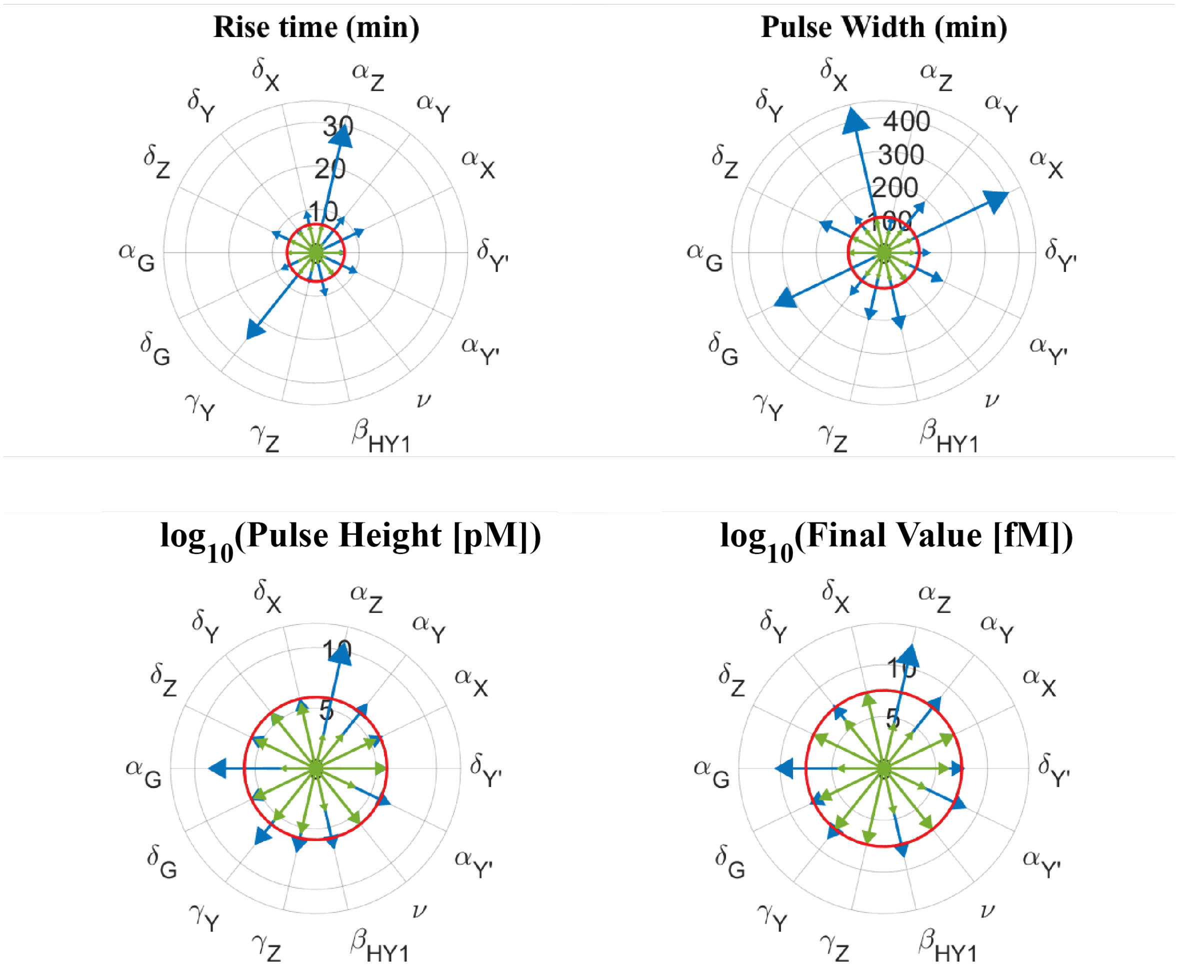

Figure S3. Local sensitivity analysis results for HY-1 Circuit. The red circle represents the nominal metric value. The blue and green arrows, respectively represent the largest and smallest metric values that can be achieved by adjusting the respective parameters. The interpretation follows the description given in the caption of Figure S1.

HY-2 Circuit

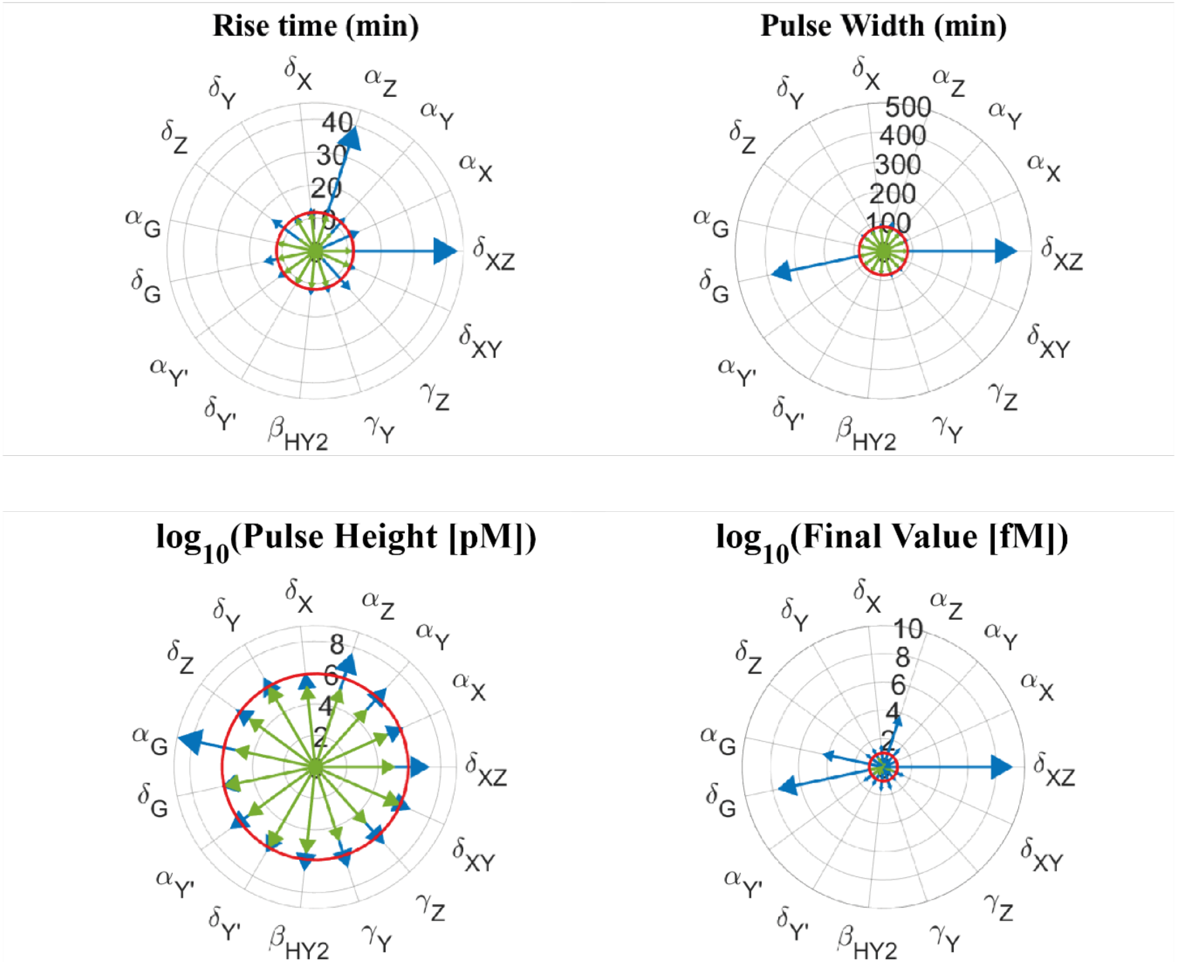

Figure S4. Local sensitivity analysis results for HY-1 Circuit. The red circle represents the nominal metric value. The blue and green arrows, respectively represent the largest and smallest metric values that can be achieved by adjusting the respective parameters. The interpretation follows the description given in the caption of Figure S1.

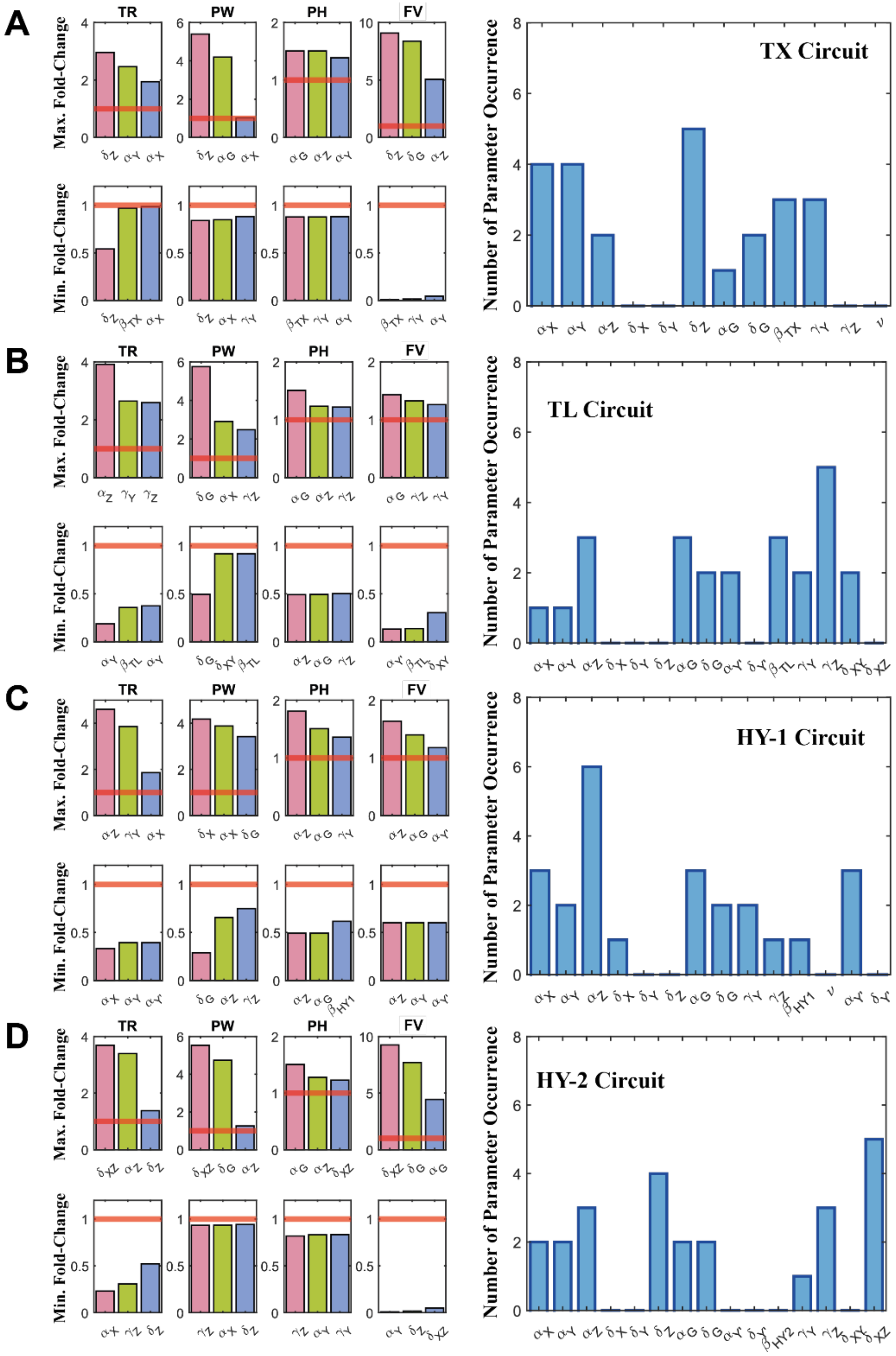

Figure S5. Local sensitivity analysis results: (Left) Relative fold-change over nominal value across the top three most influential parameters in realizing the maximum and minimum value of the metrics (Right) the associated histogram of the most influential parameters This results in a total of 24 candidate parameters. The red line indicates the nominal value for each metric: (A) TX Circuit (B) TL Circuit (C) HY-1 Circuit (D) HY-2 Circuit. The notation, TR, PW, PH and FV denote rise time, pulse width, pulse height and final value, respectively. Using the rise time metric in the TX Circuit as an illustration, the three parameters that results in the top three maximum relative fold-change from nominal value are δ_Z, α_Y and α_X which are represented by the red,

green, and blue bar graph, respectively. Likewise, the three parameters that results in the top three minimum relative fold-change from nominal value are δ_Z, β_{TX} and α_X, which are represented by the red, green, and blue bar graph, respectively. The collation of these parameters that realize the maximum and minimum relative fold-change for all four metrics can be presented with a histogram. This histogram for TX Circuit shows that δ_Z appears five times followed by α_X and α_Y, which appears four times. Discounting the parameters associated with Z mRNA and GFP, the histogram for the TX Circuit indicates that α_X and α_Y are the most impactful parameters.

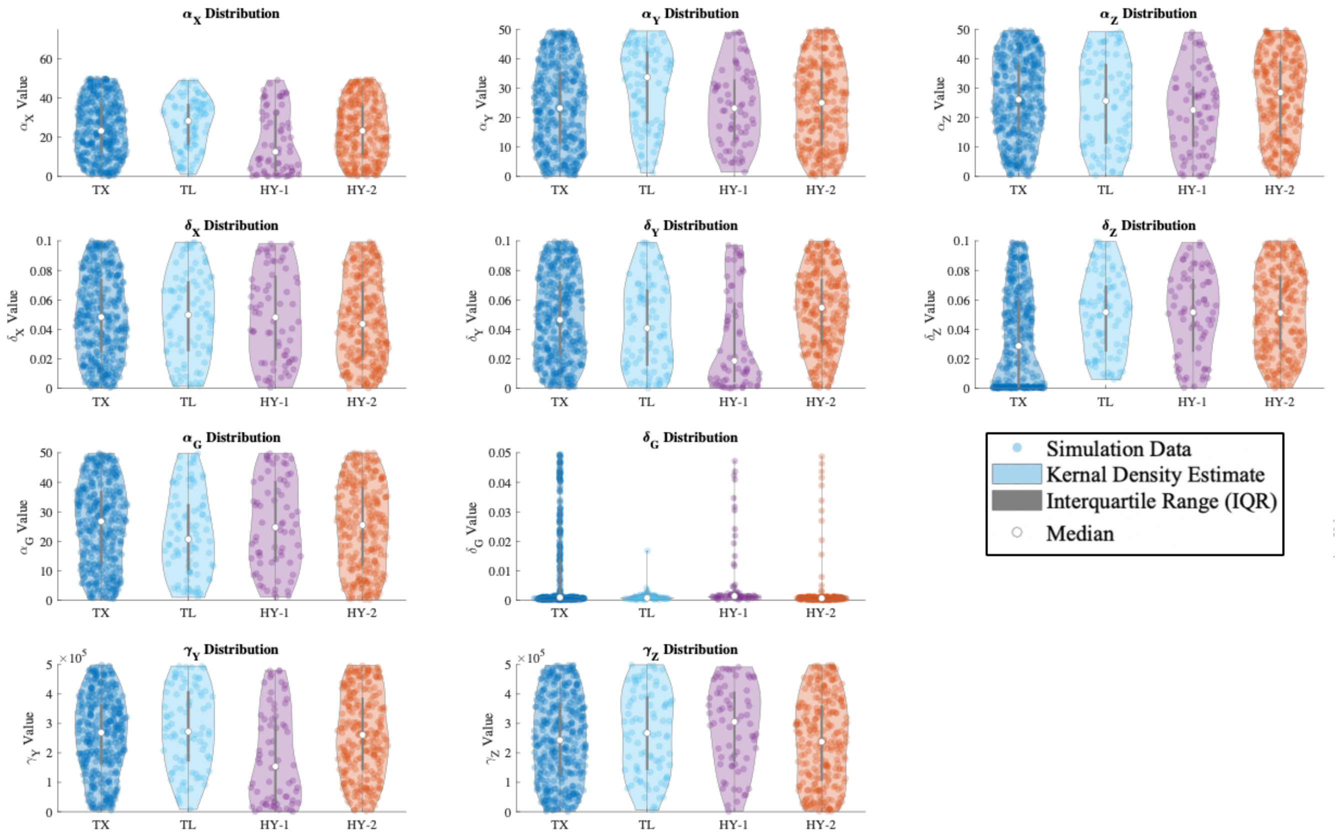

Figure S6: Global sensitivity analysis kinetic parameter distribution for parameters shared by all four circuits. Each data point represents the parameter value sampled in a specific simulation, the shaded region is the kernel density estimate, the grey box is the interquartile range of each parameter, and the clear data point represents the median value of the parameter distribution. The distribution plots represent the required kinetic parameter values for each circuit to achieve the desired pulse property. The results suggest that while the majority of the parameters do not show a prominent preference to a specific range of values, in order to achieve our specified pulse dynamics, a low δ_G value would be favored for all four circuits.

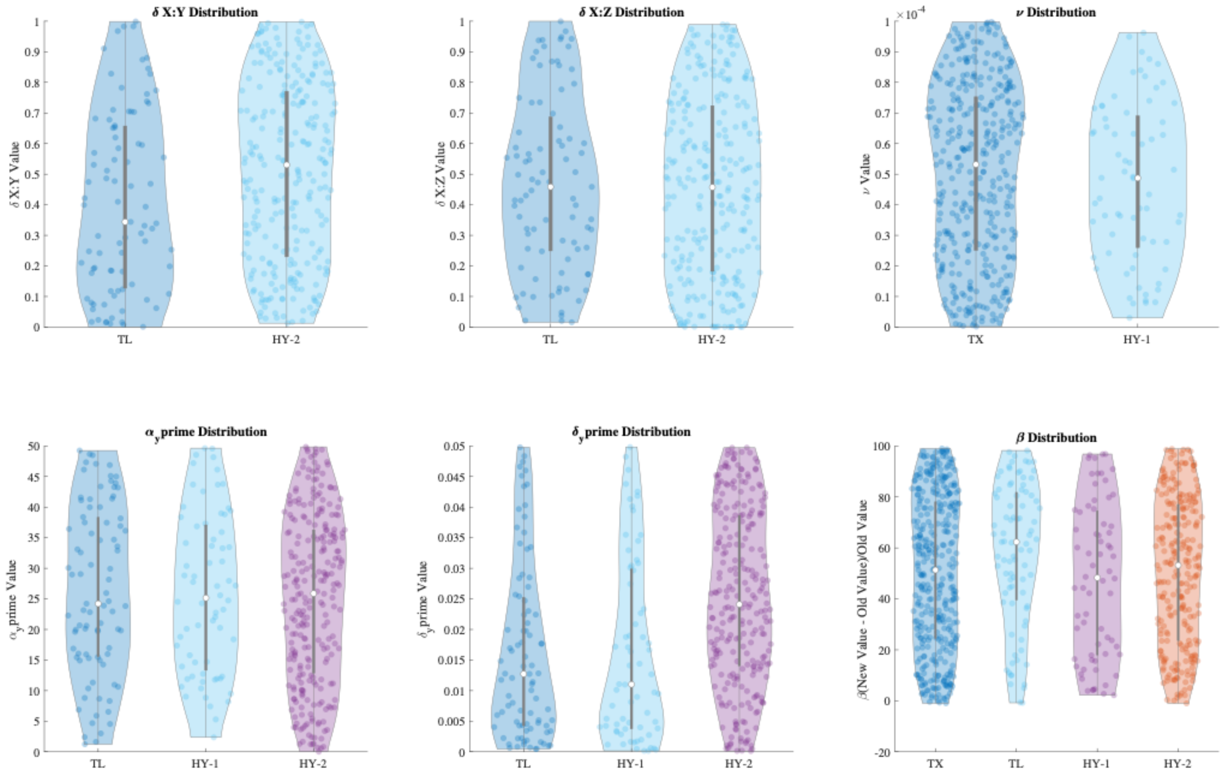

Figure S7: Global sensitivity analysis kinetic parameter distribution for parameters not shared by the circuits, including the β parameter. Due to the difference of nominal β values, the distribution was normalized by taking the difference between the nominal and the new parameter values before being divided by the nominal parameter. While the distribution indicates the achievability of the specified pulse property with any parameter value in the specified range, some parameters do show a preference to a specific parameter range. For example, for both the TL and the HY-1 Circuits, a lower $\delta_{Y'}$ value would have a higher chance of generating the desired pulse dynamics.

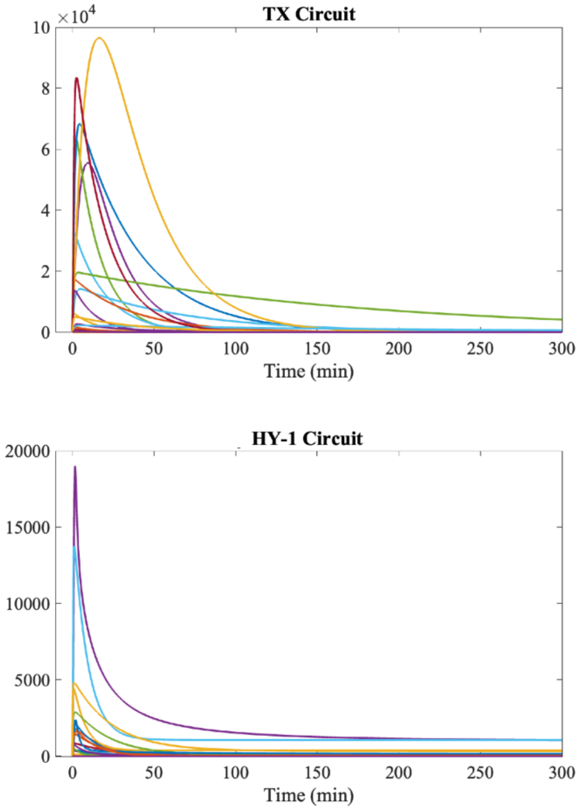

Figure S8: Simulation examples from the global sensitivity analysis that met our metric specifications. For each circuit, we randomly selected 25 simulations for visualization. Note that although all the simulations shown here have passed our metric screening, different circuits demonstrate different dynamics. The perturbations in parameter values results in varying output magnitude level and only the first 300 minutes are plotted for visualization purposes.

Supplementary Tables

Supplementary Table S1. Plasmids used in the TX Circuit. The abbreviations are as follows: T7term = T7 terminator, AmpR = ampicillin resistance gene, SpecR = spectinomycin resistance gene, KanR = kanamycin resistance gene, CmR = chloramphenicol resistance gene.

Name	Plasmid architecture
X	pT7–STAR–T7term–AmpR–pBR322 origin
Y_No TetR	J23116–TetR–T7term–SpecR–CloDF13 origin
Y_J23110 TetR	J23110–STAR Target–RBS–TetR–ASV–T7term–SpecR–CloDF13 origin
Y_J23119 TetR	J23119–STAR Target–RBS–TetR–ASV–T7term–SpecR–CloDF13 origin
Z	pT7(TetO)– STAR Target–RBS–GFPmut3b-ASV–T7term–KanR–ColA origin

Supplementary Table S2. Examples of the TX Circuit DNA plasmid sequences.

Name (architecture)	Sequence
X (pT7–STAR–T7term–(Bla Promoter)–AmpR–pBR322 origin–backbone)	TAATACGACTCACTATAGGGTGAACTGTATACATTCCCCGCAGGATGAG<br>ATGAGAACGATAGAGATGCAAAGGTAAGATGGTAGCATAACCCCTTGG<br>GGCCTCTAAACGGGTCTTGAGGGGTTTTTTGCTGAAAGGAGGAACTATA<br>TCCGGATATCCCGCAAGAGGCCCGGCAGTACCGGCATAACCAAGCCTAT<br>GCCTACAGCATCCAGGGTGACGGTGCCGAGGATGACGATGAGCGCATTG<br>TTAGATTTCATACACGGTGCCTGACTGCGTTAGCAATTTAACTGTGATAA<br>ACTACCGCATTAAAGCTTATCGATGATAAGCTGTCAAACATGAGAATTC<br>TTGAAGACGAAAGGGCCTCGTGATACGCCTATTTTTATAGGTTAATGTCA<br>TGATAATAATGGTTTCTTAGACGTCAGGTGGCACTTTTCGGGGAAATGTG<br>CGCGGAACCCCTATTTGTTTATTTTTCTAAATACATTCAAATATGTATCC<br>GCTCATGAGACAATAACCCTGATAAATGCTTCAATAATATTGAAAAAGG<br>AAGAGTATGAGTATTCAACATTTCCGTGTCGCCCTTATTCCCTTTTTTGCG<br>GCATTTTGCCTTCCTGTTTTTGCTCACCCAGAAACGCTGGTGAAAGTAAA<br>AGATGCTGAAGATCAGTTGGGTGCACGAGTGGGTTACATCGAACTGGAT<br>CTCAACAGCGGTAAGATCCTTGAGAGTTTTCGCCCCGAAGAACGTTTTCC<br>AATGATGAGCACTTTTAAAGTTCTGCTATGTGGCGCGGTATTATCCCGTG<br>TTGACGCCGGGCAAGAGCAACTCGGTCGCCGCATACACTATTCTCAGAA<br>TGACTTGGTTGAGTACTCACCAGTCACAGAAAAGCATCTTACGGATGGC<br>ATGACAGTAAGAGAATTATGCAGTGCTGCCATAACCATGAGTGATAACA<br>CTGCGGCCAACTTACTTCTGACAACGATCGGAGGACCGAAGGAGCTAAC<br>CGCTTTTTTGCACAACATGGGGGATCATGTAACTCGCCTTGATCGTTGGG<br>AACCGGAGCTGAATGAAGCCATACCAAACGACGAGCGTGACACCACGA<br>TGCCTGCAGCAATGGCAACAACGTTGCGCAAACTATTAACTGGCGAACT<br>ACTTACTCTAGCTTCCCGGCAACAATTAATAGACTGGATGGAGGCGGAT<br>AAAGTTGCAGGACCACTTCTGCGCTCGGCCCTTCCGGCTGGCTGGTTTAT<br>TGCTGATAAATCTGGAGCCGGTGAGCGTGGGTCTCGCGGTATCATTGCA<br>GCACTGGGGCCAGATGGTAAGCCCTCCCGTATCGTAGTTATCTACACGA<br>CGGGGAGTCAGGCAACTATGGATGAACGAAATAGACAGATCGCTGAGA<br>TAGGTGCCTCACTGATTAAGCATTGGTAACTGTCAGACCAAGTTTACTCA<br>TATATACTTTAGATTGATTTAAAACTTCATTTTTAATTTAAAAGGATCTA<br>GGTGAAGATCCTTTTTGATAATCTCATGACCAAAATCCCTTAACGTGAGT<br>TTTCGTTCCACTGAGCGTCAGACCCCGTAGAAAAGATCAAAGGATCTTCT<br>TGAGATCCTTTTTTTCTGCGCGTAATCTGCTGCTTGCAAACAAAAAAACC

	ACCGCTACCAGCGGTGGTTTGTTTGCCGGATCAAGAGCTACCAACTCTTT<br>TTCCGAAGGTAACTGGCTTCAGCAGAGCGCAGATACCAAATACTGTCCT<br>TCTAGTGTAGCCGTAGTTAGGCCACCACTTCAAGAACTCTGTAGCACCGC<br>CTACATACCTCGCTCTGCTAATCCTGTTACCAGTGGCTGCTGCCAGTGGC<br>GATAAGTCGTGTCTTACCGGGTTGGACTCAAGACGATAGTTACCGGATA<br>AGGCGCAGCGGTCGGGCTGAACGGGGGGTTCGTGCACACAGCCCAGCTT<br>GGAGCGAACGACCTACACCGAACTGAGATACCTACAGCGTGAGCTATGA<br>GAAAGCGCCACGCTTCCCGAAGGGAGAAAGGCGGACAGGTATCCGGTA<br>AGCGGCAGGGTCGGAACAGGAGAGCGCACGAGGGAGCTTCCAGGGGGA<br>AACGCCTGGTATCTTTATAGTCCTGTCGGGTTTCGCCACCTCTGACTTGA<br>GCGTCGATTTTTGTGATGCTCGTCAGGGGGGCGGAGCCTATGGAAAAAC<br>GCCAGCAACGCGGCCTTTTTACGGTTCCTGGCCTTTTGCTGGCCTTTTGCT<br>CACATGTTCTTTCCTGCGTTATCCCCTGATTCTGTGGATAACCGTATTACC<br>GCCTTTGAGTGAGCTGATACCGCTCGCCGCAGCCGAACGACCGAGCGCA<br>GCGAGTCAGTGAGCGAGGAAGCGGAAGAGCGCCTGATGCGGTATTTTCT<br>CCTTACGCATCTGTGCGGTATTTCACACCGCATATATGGTGCACTCTCAG<br>TACAATCTGCTCTGATGCCGCATAGTTAAGCCAGTATACACTCCGCTATC<br>GCTACGTGACTGGGTCATGGCTGCGCCCCGACACCCGCCAACACCCGCT<br>GACGCGCCCTGACGGGCTTGTCTGCTCCCGGCATCCGCTTACAGACAAG<br>CTGTGACCGTCTCCGGGAGCTGCATGTGTCAGAGGTTTTCACCGTCATCA<br>CCGAAACGCGCGAGGCAGCTGCGGTAAAGCTCATCAGCGTGGTCGTGAA<br>GCGATTCACAGATGTCTGCCTGTTCATCCGCGTCCAGCTCGTTGAGTTTC<br>TCCAGAAGCGTTAATGTCTGGCTTCTGATAAAGCGGGCCATGTTAAGGG<br>CGGTTTTTTCCTGTTTGGTCACTGATGCCTCCGTGTAAGGGGGATTTCTGT<br>TCATGGGGGTAATGATACCGATGAAACGAGAGAGGATGCTCACGATACG<br>GGTTACTGATGATGAACATGCCCGGTTACTGGAACGTTGTGAGGGTAAA<br>CAACTGGCGGTATGGATGCGGCGGGACCAGAGAAAAATCACTCAGGGT<br>CAATGCCAGCGCTTCGTTAATACAGATGTAGGTGTTCCACAGGGTAGCC<br>AGCAGCATCCTGCGATGCAGATCCGGAACATAATGGTGCAGGGCGCTGA<br>CTTCCGCGTTTCCAGACTTTACGAAACACGGAAACCGAAGACCATTCAT<br>GTTGTTGCTCAGGTCGCAGACGTTTTGCAGCAGCAGTCGCTTCACGTTCG<br>CTCGCGTATCGGTGATTCATTCTGCTAACCAGTAAGGCAACCCCGCCAGC<br>CTAGCCGGGTCCTCAACGACAGGAGCACGATCATGCGCACCCGTGGCCA<br>GGACCCAACGCTGCCCGAGATGCGCCGCGTGCGGCTGCTGGAGATGGCG<br>GACGCGATGGATATGTTCTGCCAAGGGTTGGTTTGCGCATTCACAGTTCT<br>CCGCAAGAATTGATTGGCTCCAATTCTTGGAGTGGTGAATCCGTTAGCG<br>AGGTGCCGCCGGCTTCCATTCAGGTCGAGGTGGCCCGGCTCCATGCACC<br>GCGACGCAACGCGGGGAGGCAGACAAGGTATAGGGCGGCGCCTACAAT<br>CCATGCCAACCCGTTCCATGTGCTCGCCGAGGCGGCATAAATCGCCGTG<br>ACGATCAGCGGTCCAGTGATCGAAGTTAGGCTGGTAAGAGCCGCGAGCG<br>ATCCTTGAAGCTGTCCCTGATGGTCGTCATCTACCTGCCTGGACAGCATG<br>GCCTGCAACGCGGGCATCCCGATGCCGCCGGAAGCGAGAAGAATCATA<br>ATGGGGAAGGCCATCCAGCCTCGCGTCGCGAACGCCAGCAAGACGTAGC<br>CCAGCGCGTCGGCCGCCATGCCGGCGATAATGGCCTGCTTCTCGCCGAA<br>ACGTTTGGTGGCGGGACCAGTGACGAAGGCTTGAGCGAGGGCGTGCAA<br>GATTCCGAATACCGCAAGCGACAGGCCGATCATCGTCGCGCTCCAGCGA<br>AAGCGGTCCTCGCCGAAAATGACCCAGAGCGCTGCCGGCACCTGTCCTA<br>CGAGTTGCATGATAAAGAAGACAGTCATAAGTGCGGCGACGATAGTCAT<br>GCCCCGCGCCCACCGGAAGGAGCTGACTGGGTTGAAGGCTCTCAAGGGC<br>ATCGGTCGAGATCCCGGTGCCTAATGAGTGAGCTAACTTACATTAATTGC<br>GTTGCGCTCACTGCCCGCTTTCCAGTCGGGAAACCTGTCGTGCCAGCTGC<br>ATTAATGAATCGGCCAACGCGCGGGGAGAGGCGGTTTGCGTATTGGGCG<br>CCAGGGTGGTTTTTCTTTTCACCAGTGAGACGGGCAACAGCTGATTGCCC<br>TTCACCGCCTGGCCCTGAGAGAGTTGCAGCAAGCGGTCCACGCTGGTTT<br>GCCCCAGCAGGCGAAAATCCTGTTTGATGGTGGTTAACGGCGGGATATA

	ACATGAGCTGTCTTCGGTATCGTCGTATCCCACTACCGAGATATCCGCAC<br>CAACGCGCAGCCCGGACTCGGTAATGGCGCGCATTGCGCCCAGCGCCAT<br>CTGATCGTTGGCAACCAGCATCGCAGTGGGAACGATGCCCTCATTCAGC<br>ATTTGCATGGTTTGTTGAAAACCGGACATGGCACTCCAGTCGCCTTCCCG<br>TTCCGCTATCGGCTGAATTTGATTGCGAGTGAGATATTTATGCCAGCCAG<br>CCAGACGCAGACGCGCCGAGACAGAACTTAATGGGCCCGCTAACAGCG<br>CGATTTGCTGGTGACCCAATGCGACCAGATGCTCCACGCCCAGTCGCGT<br>ACCGTCTTCATGGGAGAAAATAATACTGTTGATGGGTGTCTGGTCAGAG<br>ACATCAAGAAATAACGCCGGAACATTAGTGCAGGCAGCTTCCACAGCAA<br>TGGCATCCTGGTCATCCAGCGGATAGTTAATGATCAGCCCACTGACGCG<br>TTGCGCGAGAAGATTGTGCACCGCCGCTTTACAGGCTTCGACGCCGCTTC<br>GTTCTACCATCGACACCACCACGCTGGCACCCAGTTGATCGGCGCGAGA<br>TTTAATCGCCGCGACAATTTGCGACGGCGCGTGCAGGGCCAGACTGGAG<br>GTGGCAACGCCAATCAGCAACGACTGTTTGCCCGCCAGTTGTTGTGCCA<br>CGCGGTTGGGAATGTAATTCAGCTCCGCCATCGCCGCTTCCACTTTTTCC<br>CGCGTTTTCGCAGAAACGTGGCTGGCCTGGTTCACCACGCGGGAAACGG<br>TCTGATAAGAGACACCGGCATACTCTGCGACATCGTATAACGTTACTGG<br>TTTCACATTCACCACCCTGAATTGACTCTCTTCCGGGCGCTATCATGCCA<br>TACCGCGAAAGGTTTTGCGCCATTCGATGGTGTCCGGGATCTCGACGCTC<br>TCCCTTATGAACGTGTACGGGCTATCTGGCTTTCGTTGCGC
Y_J23110 TetR (J23110–STAR Target– RBS– TetR–ASV–T7term–SpecR– (Bla Promoter)–CloDF13 origin–backbone)	TTTACGGCTAGCTCAGTCCTAGGTACAATGCTAGCCCATCTTACCTTTGC<br>ATCTCTATCGTTCTCATCTCATCCTGCGGGGAATGTATACAGTTCATGTA<br>TATATTCCCCGCTTTTTTTTTAACCTGGCGGCAGCGCAAAAGAGAGGAGA<br>CAGAGATGTCTAGATTAGATAAAAGTAAAGTGATTAACAGCGCATTAGA<br>GCTGCTTAATGAGGTCGGAATCGAAGGTTTAACAACCCGTAAACTCGCC<br>CAGAAGCTAGGTGTAGAGCAGCCTACATTGTATTGGCATGTAAAAAATA<br>AGCGGGCTTTGCTCGACGCCTTAGCCATTGAGATGTTAGATAGGCACCA<br>TACTCACTTTTGCCCTTTAGAAGGGGAAAGCTGGCAAGATTTTTTACGTA<br>ATAACGCTAAAAGTTTTAGATGTGCTTTACTAAGTCATCGCGATGGAGC<br>AAAAGTACATTTAGGTACACGGCCTACAGAAAAACAGTATGAAACTCTC<br>GAAAATCAATTAGCCTTTTTATGCCAACAAGGTTTTTCACTAGAGAATGC<br>ATTATATGCACTCAGCGCTGTGGGGCATTTTACTTTAGGTTGCGTATTGG<br>AAGATCAAGAGCATCAAGTCGCTAAAGAAGAAAGGGAAACACCTACTA<br>CTGATAGTATGCCGCCATTATTACGACAAGCTATCGAATTATTTGATCAC<br>CAAGGTGCAGAGCCAGCCTTCTTATTCGGCCTTGAATTGATCATATGCGG<br>ATTAGAAAAACAACTTAAATGTGAAAGTGGGTCTAGGCCTGCAGCAAAC<br>GACGAAAACTACGCTGCATCAGTTTAATAAGATAAACCAGAGCGGCACG<br>GCAAGCAGAGTATACGAGATTCGGTAGCCACCGCTGAGCAATAACTAGC<br>ATAACCCCTTGGGGCCTCTAAACGGGTCTTGAGGGGTTTTTTGCTGAAAC<br>CTCAGGCATTTGAGAAGCACACGGTCACACTGCTTCCGGTAGTCAATAA<br>ACCGGTAAACCAGCAATAGACATAAGCGGCTATTTAACGACCCTGCCCT<br>GAACCGACGACCGGGTCATCGTGGCCGGATCTTGCGGCCCCTCGGCTTG<br>AACGAATTGTTAGACATTATTTGCCGACTACCTTGGTGATCTCGCCTTTC<br>ACGTAGTGGACAAATTCTTCCAACTGATCTGCGCGCGAGGCCAAGCGAT<br>CTTCTTCTTGTCCAAGATAAGCCTGTCTAGCTTCAAGTATGACGGGCTGA<br>TACTGGGCCGGCAGGCGCTCCATTGCCCAGTCGGCAGCGACATCCTTCG<br>GCGCGATTTTGCCGGTTACTGCGCTGTACCAAATGCGGGACAACGTAAG<br>CACTACATTTCGCTCATCGCCAGCCCAGTCGGGCGGCGAGTTCCATAGC<br>GTTAAGGTTTCATTTAGCGCCTCAAATAGATCCTGTTCAGGAACCGGATC<br>AAAGAGTTCCTCCGCCGCTGGACCTACCAAGGCAACGCTATGTTCTCTTG<br>CTTTTGTCAGCAAGATAGCCAGATCAATGTCGATCGTGGCTGGCTCGAA<br>GATACCTGCAAGAATGTCATTGCGCTGCCATTCTCCAAATTGCAGTTCGC<br>GCTTAGCTGGATAACGCCACGGAATGATGTCGTCGTGCACAACAATGGT<br>GACTTCTACAGCGCGGAGAATCTCGCTCTCTCCAGGGGAAGCCGAAGTT<br>TCCAAAAGGTCGTTGATCAAAGCTCGCCGCGTTGTTTCATCAAGCCTTAC

	GGTCACCGTAACCAGCAAATCAATATCACTGTGTGGCTTCAGGCCGCCA<br>TCCACTGCGGAGCCGTACAAATGTACGGCCAGCAACGTCGGTTCGAGAT<br>GGCGCTCGATGACGCCAACTACCTCTGATAGTTGAGTCGATACTTCGGC<br>GATCACCGCTTCCCTCATACTCTTCCTTTTTCAATATTATTGAAGCATTTA<br>TCAGGGTTATTGTCTCATGAGCGGATACATATTTGAATGTATTTAGAAAA<br>ATAAACAAATAGCTAGCTCACTCGGTCGCTACGCTCCGGGCGTGAGACT<br>GCGGCGGGCGCTGCGGACACATACAAAGTTACCCACAGATTCCGTGGAT<br>AAGCAGGGGACTAACATGTGAGGCAAAACAGCAGGGCCGCGCCGGTGG<br>CGTTTTTCCATAGGCTCCGCCCTCCTGCCAGAGTTCACATAAACAGACGC<br>TTTTCCGGTGCATCTGTGGGAGCCGTGAGGCTCAACCATGAATCTGACA<br>GTACGGGCGAAACCCGACAGGACTTAAAGATCCCCACCGTTTCCGGCGG<br>GTCGCTCCCTCTTGCGCTCTCCTGTTCCGACCCTGCCGTTTACCGGATACC<br>TGTTCCGCCTTTCTCCCTTACGGGAAGTGTGGCGCTTTCTCATAGCTCAC<br>ACACTGGTATCTCGGCTCGGTGTAGGTCGTTCGCTCCAAGCTGGGCTGTA<br>AGCAAGAACTCCCCGTTCAGCCCGACTGCTGCGCCTTATCCGGTAACTGT<br>TCACTTGAGTCCAACCCGGAAAAGCACGGTAAAACGCCACTGGCAGCAG<br>CCATTGGTAACTGGGAGTTCGCAGAGGATTTGTTTAGCTAAACACGCGG<br>TTGCTCTTGAAGTGTGCGCCAAAGTCCGGCTACACTGGAAGGACAGATT<br>TGGTTGCTGTGCTCTGCGAAAGCCAGTTACCACGGTTAAGCAGTTCCCCA<br>ACTGACTTAACCTTCGATCAAACCACCTCCCCAGGTGGTTTTTTCGTTTA<br>CAGGGCAAAAGATTACGCGCAGAAAAAAAGGATCTCAAGAAGATCCTT<br>TGATCTTTTCTACTGAACCGCTCTAGATTTCAGTGCAATTTATCTCTTCAA<br>ATGTAGCACCTGAAGTCAGCCCCATACGATATAAGTTGTAATTCTCATGT<br>TAGTCATGCCCCGCGCCCACCGGAAGGAGCTGACTGGGTTGAAGGCTCT<br>CAAGGGCATCGGTCGAGATCCCGGTGCCTAATGAGTGAGCTAACTTACA<br>TTAATTGCGTTGCGCTCACTGCCCGCTTTCCAGTCGGGAAACCTGTCGTG<br>CCAGCTGCATTAATGAATCGGCCAACGCGCGGGGAGAGGCGGTTTGCGT<br>ATTGGGCGCCAGGGTGGTTTTTCTTTTCACCAGTGAGACGGGCAACAGCT<br>GATTGCCCTTCACCGCCTGGCCCTGAGAGAGTTGCAGCAAGCGGTCCAC<br>GCTGGTTTGCCCCAGCAGGCGAAAATCCTGTTTGATGGTGGTTAACGGC<br>GGGATATAACATGAGCTGTCTTCGGTATCGTCGTATCCCACTACCGAGAT<br>GTCCGCACCAACGCGCAGCCCGGACTCGGTAATGGCGCGCATTGCGCCC<br>AGCGCCATCTGATCGTTGGCAACCAGCATCGCAGTGGGAACGATGCCCT<br>CATTCAGCATTTGCATGGTTTGTTGAAAACCGGACATGGCACTCCAGTCG<br>CCTTCCCGTTCCGCTATCGGCTGAATTTGATTGCGAGTGAGATATTTATG<br>CCAGCCAGCCAGACGCAGACGCGCCGAGACAGAACTTAATGGGCCCGCT<br>AACAGCGCGATTTGCTGGTGACCCAATGCGACCAGATGCTCCACGCCCA<br>GTCGCGTACCGTCTTCATGGGAGAAAATAATACTGTTGATGGGTGTCTG<br>GTCAGAGACATCAAGAAATAACGCCGGAACATTAGTGCAGGCAGCTTCC<br>ACAGCAATGGCATCCTGGTCATCCAGCGGATAGTTAATGATCAGCCCAC<br>TGACGCGTTGCGCGAGAAGATTGTGCACCGCCGCTTTACAGGCTTCGAC<br>GCCGCTTCGTTCTACCATCGACACCACCACGCTGGCACCCAGTTGATCGG<br>CGCGAGATTTAATCGCCGCGACAATTTGCGACGGCGCGTGCAGGGCCAG<br>ACTGGAGGTGGCAACGCCAATCAGCAACGACTGTTTGCCCGCCAGTTGT<br>TGTGCCACGCGGTTGGGAATGTAATTCAGCTCCGCCATCGCCGCTTCCAC<br>TTTTTCCCGCGTTTTCGCAGAAACGTGGCTGGCCTGGTTCACCACGCGGG<br>AAACGGTCTGATAAGAGACACCGGCATACTCTGCGACATCGTATAACGT<br>TACTGGTTTCACATTCACCACCCTGAATTGACTCTCTTCCGGGCGCTATC<br>ATGCCATACCGCGAAAGGTTTTGCGCCATTCGATGGTGTCCGGGATCTCG<br>ACGCTCTCCCTTATGAGTGATAGCCGTTTGTCTGGTGTCTACGCCGCGC
Z (pT7–TetO – STAR Target–RBS–GFPmut3b-ASV–T7term–KanR–(Bla Promoter)–ColA origin–backbone)	TAATACGACTCACTATAGGTCTATCATTGATAGGGTTTCCATCTTACCTT<br>TGCATCTCTATCGTTCTCATCTCATCCTGCGGGGAATGTATACAGTTCAT<br>GTATATATTCCCCGCTTTTTTTTTAACCTGGCGGCAGCGCAAAAGAGAGG<br>AGACAGAGATGCGTAAAGGAGAAGAACTTTTCACTGGAGTTGTCCCAAT<br>TCTTGTTGAATTAGATGGTGATGTTAATGGGCACAAATTTTCTGTCAGTG

GAGAGGGTGAAGGTGATGCAACATACGGAAAACTTACCCTTAAATTTAT
TTGCACTACTGGAAAACTACCTGTTCCGTGGCCAACACTTGTCACTACTT
TCGGTTATGGTGTTCAATGCTTTGCGAGATACCCAGATCACATGAAACA
GCATGACTTTTTCAAGAGTGCCATGCCCGAAGGTTACGTACAGGAAAGA
ACTATATTTTTCAAAGATGACGGGAACTACAAGACACGTGCTGAAGTCA
AGTTTGAAGGTGATACCCTTGTTAATAGAATCGAGTTAAAAGGTATTGA
TTTTAAAGAAGATGGAAACATTCTTGGACACAAATTGGAATACAACTAT
AACTCACACAATGTATACATCATGGCAGACAAACAAAAGAATGGAATCA
AAGTTAACTTCAAAATTAGACACAACATTGAAGATGGAAGCGTTCAACT
AGCAGACCATTATCAACAAAATACTCCGATTGGCGATGGCCCTGTCCTTT
TACCAGACAACCATTACCTGTCCACACAATCTGCCCTTTCGAAAGATCCC
AACGAAAAGAGAGACCACATGGTCCTTCTTGAGTTTGTAACCGCTGCTG
GGATTACACATGGCATGGATGAACTATACAAAAGGCCTGCAGCAAACGA
CGAAAACTACGCTGCATCAGTTTAATAAGATAAACCAGAGCGGCACGGC
AAGCAGAGTATACGAGATTCGGTAGCCACCGCTGAGCAATAACTAGCAT
AACCCCTTGGGGCCTCTAAACGGGTCTTGAGGGGTTTTTTGCTGAAACCT
CAGGCATTTGAGAAGCACACGGTCACACTGCTTCCGGTAGTCAATAAAC
CGGTAAACCAGCAATAGACATAAGCGGCTATTTAACGACCCTGCCCTGA
ACCGACGACAAGCTGACGACCGGGTCTCCGCAAGTGGCACTTTTCGGGG
AAATGTGCGCGGAACCCCTATTTGTTTATTTTTCTAAATACATTCAAATA
TGTATCCGCTCATGAATTAATTCTTAGAAAAACTCATCGAGCATCAAATG
AAACTGCAATTTATTCATATCAGGATTATCAATACCATATTTTTGAAAAA
GCCGTTTCTGTAATGAAGGAGAAAACTCACCGAGGCAGTTCCATAGGAT
GGCAAGATCCTGGTATCGGTCTGCGATTCCGACTCGTCCAACATCAATAC
AACCTATTAATTTCCCCTCGTCAAAAATAAGGTTATCAAGTGAGAAATC
ACCATGAGTGACGACTGAATCCGGTGAGAATGGCAAAAGTTTATGCATT
TCTTTCCAGACTTGTTCAACAGGCCAGCCATTACGCTCGTCATCAAAATC
ACTCGCATCAACCAAACCGTTATTCATTCGTGATTGCGCCTGAGCGAGAC
GAAATACGCGGTCGCTGTTAAAAGGACAATTACAAACAGGAATCGAATG
CAACCGGCGCAGGAACACTGCCAGCGCATCAACAATATTTTCACCTGAA
TCAGGATATTCTTCTAATACCTGGAATGCTGTTTTCCCGGGGATCGCAGT
GGTGAGTAACCATGCATCATCAGGAGTACGGATAAAATGCTTGATGGTC
GGAAGAGGCATAAATTCCGTCAGCCAGTTTAGTCTGACCATCTCATCTGT
AACATCATTGGCAACGCTACCTTTGCCATGTTTCAGAAACAACTCTGGCG
CATCGGGCTTCCCATACAATCGATAGATTGTCGCACCTGATTGCCCGACA
TTATCGCGAGCCCATTTATACCCATATAAATCAGCATCCATGTTGGAATT
TAATCGCGGCCTAGAGCAAGACGTTTCCCGTTGAATATGGCTCATACTCT
TCCTTTTTCAATATTATTGAAGCATTTATCAGGGTTATTGTCTCATGAGCG
GATACATATTTGAATGTATTTAGAAAAATAAACAAATAGGCATGCTAGC
GCAGAAACGTCCTAGAAGATGCCAGGAGGATACTTAGCAGAGAGACAA
TAAGGCCGGAGCGAAGCCGTTTTTCCATAGGCTCCGCCCCCCTGACGAA
CATCACGAAATCTGACGCTCAAATCAGTGGTGGCGAAACCCGACAGGAC
TATAAAGATACCAGGCGTTTCCCCCTGATGGCTCCCTCTTGCGCTCTCCT
GTTCCCGTCCTGCGGCGTCCGTGTTGTGGTGGAGGCTTTACCCAAATCAC
CACGTCCCGTTCCGTGTAGACAGTTCGCTCCAAGCTGGGCTGTGTGCAAG
AACCCCCCGTTCAGCCCGACTGCTGCGCCTTATCCGGTAACTATCATCTT
GAGTCCAACCCGGAAAGACACGACAAAACGCCACTGGCAGCAGCCATT
GGTAACTGAGAATTAGTGGATTTAGATATCGAGAGTCTTGAAGTGGTGG
CCTAACAGAGGCTACACTGAAAGGACAGTATTTGGTATCTGCGCTCCAC
TAAAGCCAGTTACCAGGTTAAGCAGTTCCCCAACTGACTTAACCTTCGAT
CAAACCGCCTCCCCAGGCGGTTTTTTCGTTTACAGAGCAGGAGATTACG
ACGATCGTAAAAGGATCTCAAGAAGATCCTTTACGGATTCCCGACACCA
TCACTCTAGATTTCAGTGCAATTTATCTCTTCAAATGTAGCACCTGAAGT
CAGCCCCATACGATATAAGTTGTAATTCTCATGTTAGTCATGCCCCGCGC
CCACCGGAAGGAGCTGACTGGGTTGAAGGCTCTCAAGGGCATCGGTCGA

	GATCCCGGTGCCTAATGAGTGAGCTAACTTACATTAATTGCGTTGCGCTC<br>ACTGCCCGCTTTCCAGTCGGGAAACCTGTCGTGCCAGCTGCATTAATGAA<br>TCGGCCAACGCGCGGGGAGAGGCGGTTTGCGTATTGGGCGCCAGGGTGG<br>TTTTTCTTTTCACCAGTGAGACGGGCAACAGCTGATTGCCCTTCACCGCC<br>TGGCCCTGAGAGAGTTGCAGCAAGCGGTCCACGCTGGTTTGCCCCAGCA<br>GGCGAAAATCCTGTTTGATGGTGGTTAACGGCGGGATATAACATGAGCT<br>GTCTTCGGTATCGTCGTATCCCACTACCGAGATGTCCGCACCAACGCGCA<br>GCCCGGACTCGGTAATGGCGCGCATTGCGCCCAGCGCCATCTGATCGTT<br>GGCAACCAGCATCGCAGTGGGAACGATGCCCTCATTCAGCATTTGCATG<br>GTTTGTTGAAAACCGGACATGGCACTCCAGTCGCCTTCCCGTTCCGCTAT<br>CGGCTGAATTTGATTGCGAGTGAGATATTTATGCCAGCCAGCCAGACGC<br>AGACGCGCCGAGACAGAACTTAATGGGCCCGCTAACAGCGCGATTTGCT<br>GGTGACCCAATGCGACCAGATGCTCCACGCCCAGTCGCGTACCGTCTTC<br>ATGGGAGAAAATAATACTGTTGATGGGTGTCTGGTCAGAGACATCAAGA<br>AATAACGCCGGAACATTAGTGCAGGCAGCTTCCACAGCAATGGCATCCT<br>GGTCATCCAGCGGATAGTTAATGATCAGCCCACTGACGCGTTGCGCGAG<br>AAGATTGTGCACCGCCGCTTTACAGGCTTCGACGCCGCTTCGTTCTACCA<br>TCGACACCACCACGCTGGCACCCAGTTGATCGGCGCGAGATTTAATCGC<br>CGCGACAATTTGCGACGGCGCGTGCAGGGCCAGACTGGAGGTGGCAAC<br>GCCAATCAGCAACGACTGTTTGCCCGCCAGTTGTTGTGCCACGCGGTTGG<br>GAATGTAATTCAGCTCCGCCATCGCCGCTTCCACTTTTTCCCGCGTTTTCG<br>CAGAAACGTGGCTGGCCTGGTTCACCACGCGGGAAACGGTCTGATAAGA<br>GACACCGGCATACTCTGCGACATCGTATAACGTTACTGGTTTCACATTCA<br>CCACCCTGAATTGACTCTCTTCCGGGCGCTATCATGCCATACCGCGAAAG<br>GTTTTGCGCCATTCGATGGTGTCCGGGATCTCGACGCTCTCCCTTATGAA<br>GTCTAACGCTGCTCTGGGCTAACTGTC

Supplementary Table S3. Promoter sequences used in the TX Circuit. Plasmid sequences can be constructed by replacing the yellow region in the example plasmids in **Table S2** with the yellow region indicated here.

Name	Sequence
pT7	TAATACGACTCACTATAGG
J23116	TTGACAGCTAGCTCAGTCCTAGGGACTATGCTAGC
J23110	TTTACGGCTAGCTCAGTCCTAGGTACAATGCTAGC
J23119	TTGACAGCTAGCTCAGTCCTAGGTATAATACTAGT

Supplementary Table S4. Metric quantification of the TX Circuit experimental validation in Figure 8A. Note that these values are estimated manually, given the limited amount of data points from the experiments.

	RT (min)	PW (min)	PH(a.u.)	FV (a.u.)
aTc 100 ng/ml J23119 TetR	50	100	574	375
aTc 50 ng/ml J23119 TetR	40	40	350	200
aTc 20 ng/ml J23110 TetR	40	50	500	300
